# Analysis of banana plant health using machine learning techniques

**DOI:** 10.1038/s41598-024-63930-y

**Published:** 2024-07-01

**Authors:** Joshva Devadas Thiagarajan, Siddharaj Vitthal Kulkarni, Shreyas Anil Jadhav, Ayush Ashish Waghe, S. P. Raja, Sivakumar Rajagopal, Harshit Poddar, Shamala Subramaniam

**Affiliations:** 1 School of Computer Science and Engineering, Vellore Institute of Technology, Vellore, Tamil Nadu 632014 India; 2 School of Electronics Engineering, Vellore Institute of Technology, Vellore, Tamil Nadu 632014 India; 3 Department of Communication Technology and Networks, Universiti Putra Malaysia, Selangor, Malaysia

**Keywords:** Banana industry, Disease detection, Agricultural farming, Machine learning algorithms, Banana leaf diseases, Automated systems, Plant sciences, Computational biology and bioinformatics

## Abstract

The Indian economy is greatly influenced by the Banana Industry, necessitating advancements in agricultural farming. Recent research emphasizes the imperative nature of addressing diseases that impact Banana Plants, with a particular focus on early detection to safeguard production. The urgency of early identification is underscored by the fact that diseases predominantly affect banana plant leaves. Automated systems that integrate machine learning and deep learning algorithms have proven to be effective in predicting diseases. This manuscript examines the prediction and detection of diseases in banana leaves, exploring various diseases, machine learning algorithms, and methodologies. The study makes a contribution by proposing two approaches for improved performance and suggesting future research directions. In summary, the objective is to advance understanding and stimulate progress in the prediction and detection of diseases in banana leaves. The need for enhanced disease identification processes is highlighted by the results of the survey. Existing models face a challenge due to their lack of rotation and scale invariance. While algorithms such as random forest and decision trees are less affected, initially convolutional neural networks (CNNs) is considered for disease prediction. Though the Convolutional Neural Network models demonstrated impressive accuracy in many research but it lacks in invariance to scale and rotation. Moreover, it is observed that due its inherent design it cannot be combined with feature extraction methods to identify the banana leaf diseases. Due to this reason two alternative models that combine ANN with scale-invariant Feature transform (SIFT) model or histogram of oriented gradients (HOG) combined with local binary patterns (LBP) model are suggested. The first model ANN with SIFT identify the disease by using the activation functions to process the features extracted by the SIFT by distinguishing the complex patterns. The second integrate the combined features of HOG and LBP to identify the disease thus by representing the local pattern and gradients in an image. This paves a way for the ANN to learn and identify the banana leaf disease. Moving forward, exploring datasets in video formats for disease detection in banana leaves through tailored machine learning algorithms presents a promising avenue for research.

## Introduction

Agriculture functions as a fundamental pillar of any nation's economy, and within this sector, the cultivation of bananas holds notable significance. In the fiscal year 2019, bananas made a substantial contribution of 346 billion Indian rupees to India's economy, thereby solidifying its position as a critical food crop. Nevertheless, the industry encounters formidable challenges presented by diseases such as Black Sigatoka, Panama wilt, and Mosaic, resulting in reduced yields and impacting both farmers and the national economy. This paper underscores the imperative necessity for innovative methods in banana production to mitigate losses caused by diseases, with particular emphasis on early detection through the integration of cutting-edge technologies. The research highlights the role of machine learning and deep learning algorithms in the identification and classification of banana diseases. The process encompasses a series of steps, including image acquisition, pre-processing, segmentation, feature extraction, selection, and leaf classification. Multiple algorithms, including Convolutional Neural Network (CNN) for feature extraction, Support Vector Machine (SVM) for classification, Local Binary Pattern (LBP) for feature extraction, and a combination of SVM and K-Nearest Neighbour (KNN) for classification, are explored for their efficacy. However, a comprehensive examination reveals certain limitations, particularly in terms of sensitivity to scale and rotation. Overcoming these constraints is crucial to ensure the robustness of the models.

The motivation behind this study lies in the objective of contributing to the early detection of banana leaf diseases using Machine Learning and Deep Learning Algorithms. Given India's leading role in global banana production, the paper seeks to address critical inquiries, such as the existence of proposed algorithms for early detection, the selection of machine learning for prediction, the potential for devising novel models, issues in current models, practical applications for the benefit of farmers, and the necessity of pre-processing raw data for classification. The significance of the study extends to its contributions, providing a comprehensive overview of technological techniques employed in identifying banana leaf diseases, featuring a comparative analysis of various approaches. Notably, it serves as a pioneering contribution by acquainting readers with diverse approaches used for banana leaf disease identification, bridging a gap in the existing research literature.

Banana leaves disease detection uses fused features such as SIFT or LBP can enhance the accuracy and robustness. By combining the complementary information provided by these two feature extraction methods, it is possible to capture both the local texture and global structure of the leaves, resulting in a more comprehensive representation of disease patterns. Theses fused features are proposed as two models in this paper such as ANN combined with SIFT or HOG + LBP. The authors consider ANN + SIFT model for implementation.

The proposed model HOG + LBP + ANN, initially utilize the pre-processed data for extracting the features. These extracted features are flattened into ID array to ensure compatibility with ANN. Further, ANN uses its hyperparameters determined through cross validation for classification and disease prediction. The combined effort optimizes the neural network architectures and parameters and thus excels in recognizing leaf diseases.

Moreover, the implementation model ANN + SIFT uses SIFT to extract the features and these features are flattened to 1D array for compatibility with ANN. Finally ANN utilized it’s the activation function and regularization techniques on the trained dataset to prevent overfitting. This pipelined effort can lead to improved performance in disease prediction, diagnosis and management strategies for banana crops.

Further research on the application of fused features in other crop diseases can help to determine the effectiveness and generalizability of this approach. By exploring the use of different feature extraction methods and evaluating their impact on disease detection and management, we can expand the potential applications of fused features in agriculture and contribute to more accurate and efficient crop disease diagnosis across various crops.

In furnishing essential background information, the paper explores the distinctive growth process of the banana plant, which does not originate from a seed but instead from a bulb or rhizome. Various diseases affecting different parts of the plant, such as leaves, stems, roots, flowers, suckers, and fruit, are highlighted, setting the stage for comprehending the challenges encountered in banana production. This comprehensive overview lays the foundation for the subsequent exploration of innovative technologies in banana production, with a specific focus on machine learning and deep learning algorithms for disease detection. In conclusion, this paper aims to make a significant contribution to the field of banana production by advocating for advanced technological solutions and innovative approaches to disease detection, ultimately benefiting farmers and strengthening the resilience of the industry.

## Banana leaf diseases and their significance

Banana plants exhibit susceptibility to a variety of diseases that possess the potential to exert a significant impact on their well-being, productivity, and overall agricultural output. It is of utmost importance to promptly detect and effectively manage these diseases in order to sustain banana cultivation and ensure a consistent supply of this indispensable crop. Among the plethora of banana leaf diseases, the Fusarium Oxysporum fungusinduced Panama wilt emerges as an exceptionally noteworthy menace. Banana leaf diseases encompass a diverse spectrum of maladies that afflict distinct segments of the plant, including the leaves, stems, roots, and fruit. These diseases pose a substantial peril to banana cultivation, frequently resulting in diminished yields and financial losses for farmers. The implementation of effective control measures hinges upon prompt and accurate detection. Noteworthy examples of banana leaf diseases include Black Sigatoka, Mosaic disease, Anthracnose, Xanthomonas wilt, and Fusarium wilt, commonly referred to as Panama disease.

### Panama wilt/*Fusarium* Wilt

**Figure 1:**
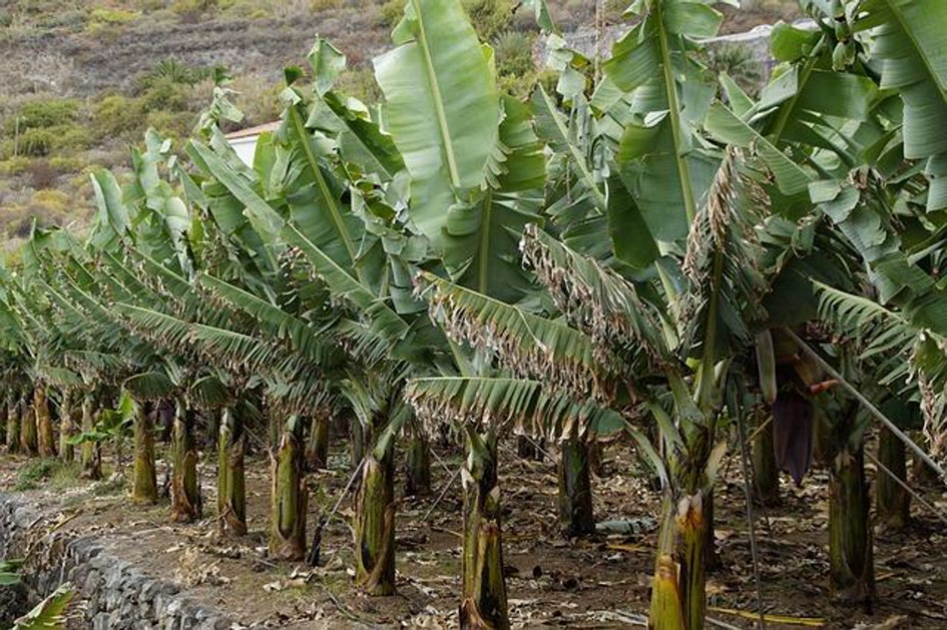
Panama wilt disease.

Panama wilt (in Fig. 1), more commonly referred to as Fusarium wilt disease, is a condition that arises due to the presence of the fungus Fusarium Oxysporum. This particular pathogen predominantly targets the roots of the banana plant and becomes evident through the observable symptoms that manifest on the leaves. Infection typically originates from rhizomes that have been contaminated. The initial indications of Panama wilt become noticeable on the banana leaves themselves. Infected leaves exhibit a characteristic transformation, commencing with a discolouration that ranges from brown to dark red. The edges of older leaves become yellowed, thereby creating a visual contrast. As the disease progresses, usually within a span of one or two months, the infected leaves begin to sag, eventually drooping until they reach the pseudo stem. In the final stages, the leaves take on a purple or black hue, thereby indicating severe harm to the plant. The detection of Panama wilt is of utmost importance for the overall health of banana plants and the sustainability of the crop. One of the primary methods for detecting this disease involves regular observation of the leaves. Early symptoms, such as discoloration and yellowing, should serve as a warning sign to farmers, prompting them to take immediate action. Swift intervention is crucial in order to mitigate the spread of the disease, as Fusarium wilt has the potential to cause significant losses in banana yields if left unaddressed^1–4^.

### Bract Mosaic

**Figure 2:**
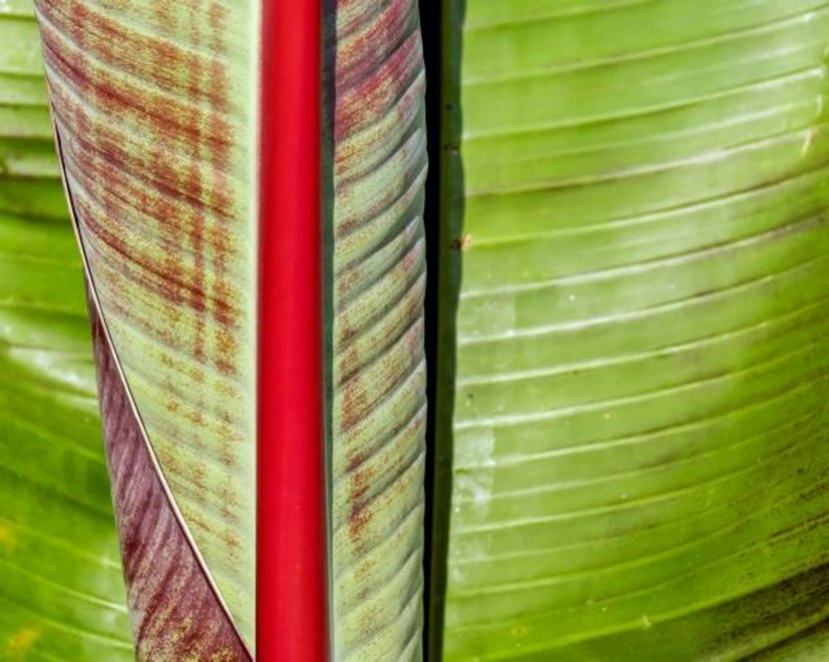
Bract Mosaic disease^7^.

The Bract Mosaic (in Fig. 2) virus is responsible for the occurrence of Bract Mosaic disease, which poses a significant threat to banana plants at different stages of growth, including vegetative, flowering, and fruiting phases. The manifestation of this viral infection is visually evident through a distinct mottled appearance in the plants, indicating an underlying pathological condition^5,6^. The origin of these viruses can be traced back to various lineages that are unrelated, which further emphasizes the adaptability and widespread impact of Bract Mosaic disease. The initial symptoms of Bract Mosaic disease are characterized by the presence of streaks of green or red-brown color on the leaves, creating a noticeable contrast to the natural state of the plant. Notably, the stalks of leaves also display spindle-shaped lesions, contributing to the unique appearance of infected plants. In some cases, these lesions may extend to the midribs of the leaves, broadening the range of visual indicators. As the disease progresses, the leaves undergo a transformative process, turning brown and ultimately succumbing to death. This sequence of symptoms signifies the severe impact of the Bract Mosaic virus on banana plants.

The detection of Bract Mosaic disease relies on careful observation of these characteristic symptoms. The mottled appearance, streaks of color, spindle-shaped lesions, and browning of leaves serve as crucial indicators for early identification. Swift detection is crucial for implementing control measures and preventing the further spread of the virus within banana plantations. Addressing Bract Mosaic disease is not only important for the visual aesthetics of the plantation but also has broader implications. The mottled appearance compromises the overall appeal of banana plants, thereby impacting their market value. Moreover, the consequences of the viral infection can lead to significant losses in yield, affecting the economic viability of banana cultivation. Preserving the health of banana plants is of utmost importance as the infected leaves, turning brown and eventually dying, contribute to a decline in the vitality of the plant. In essence, understanding and effectively managing Bract Mosaic disease are imperative for sustaining the health and productivity of banana crops in the face of this formidable viral menace^7^.

### Black Sigatoka

**Figure 3:**
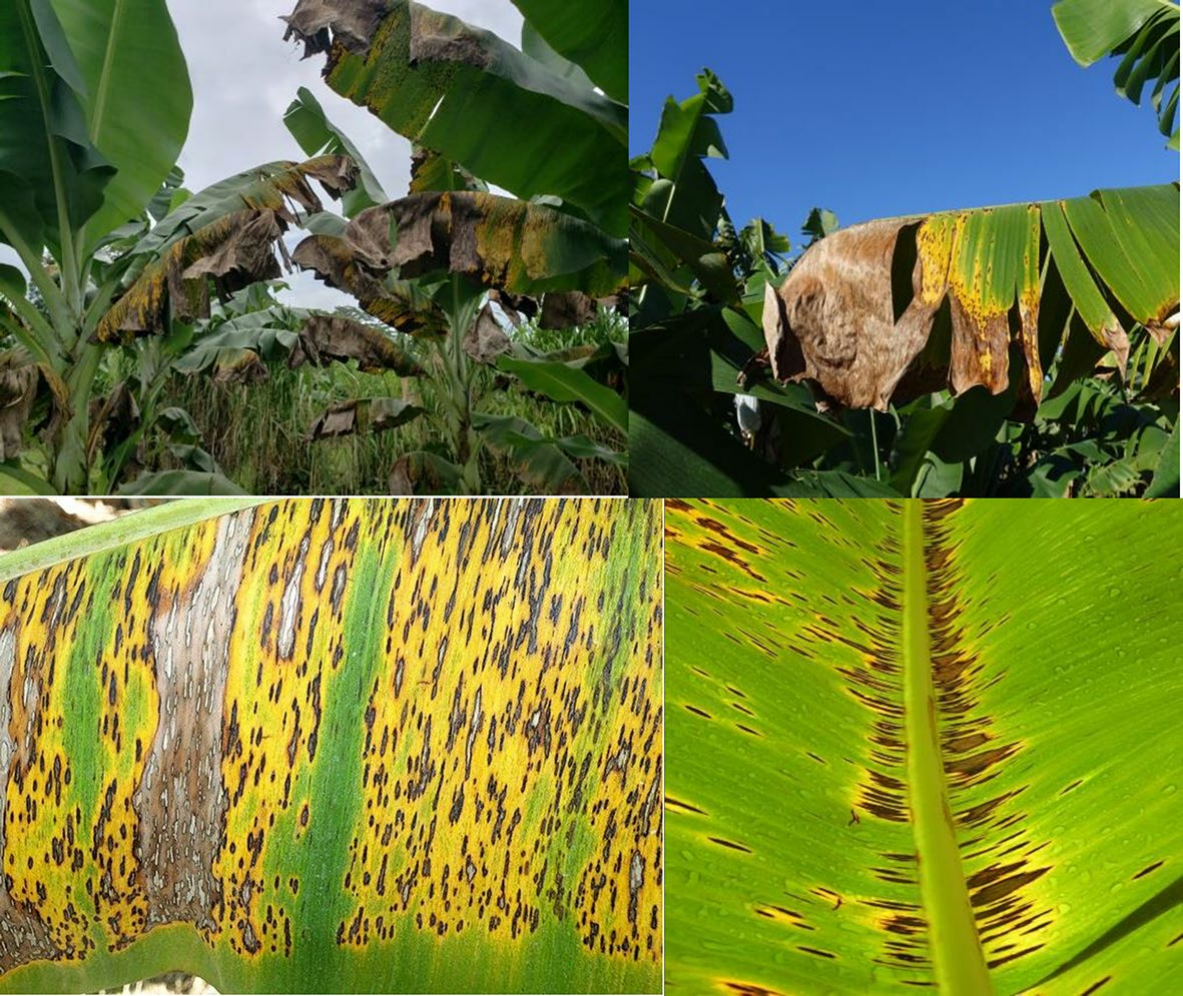
Black Sigatoka.

Black Sigatoka (in Fig. 3) caused primarily by Phyllosticpina Nusarus, poses a significant challenge to banana plants, greatly affecting both fruit yield and overall crop quality. This disease, primarily observed underneath the leaves, presents itself in manners that can compromise the economic sustainability of banana cultivation. The pernicious nature of Black Sigatoka is evident in its negative impact on fruit yield and the premature ripening of fruit clusters. The infected leaves ominously contribute to reduced productivity, resulting in mixed ripening and premature dropping of fruit clusters. The economic consequences of Black Sigatoka are substantial, prioritizing the effective management of this disease for banana cultivators^8,9^. The visual indications of Black Sigatoka commence with the emergence of spots on the leaves. These spots exhibit gray or light brown centers enclosed by a dark brown or black border, as illustrated in Fig. 3. As the disease progresses, wide bands develop on both sides of the midrib, the prominent vein along the center of a leaf. The distinct appearance of these bands serves as a visual indicator of the severity of the infection. Early detection of Black Sigatoka is crucial for implementing timely control measures. During the initial stage, the drying of the back of the midrib is a key symptom that aids in identifying the disease. This proactive approach is essential in preventing the further spread of the disease and minimizing its impact on banana plantations^10– 12^.

### Bunchy top virus

**Figure 4:**
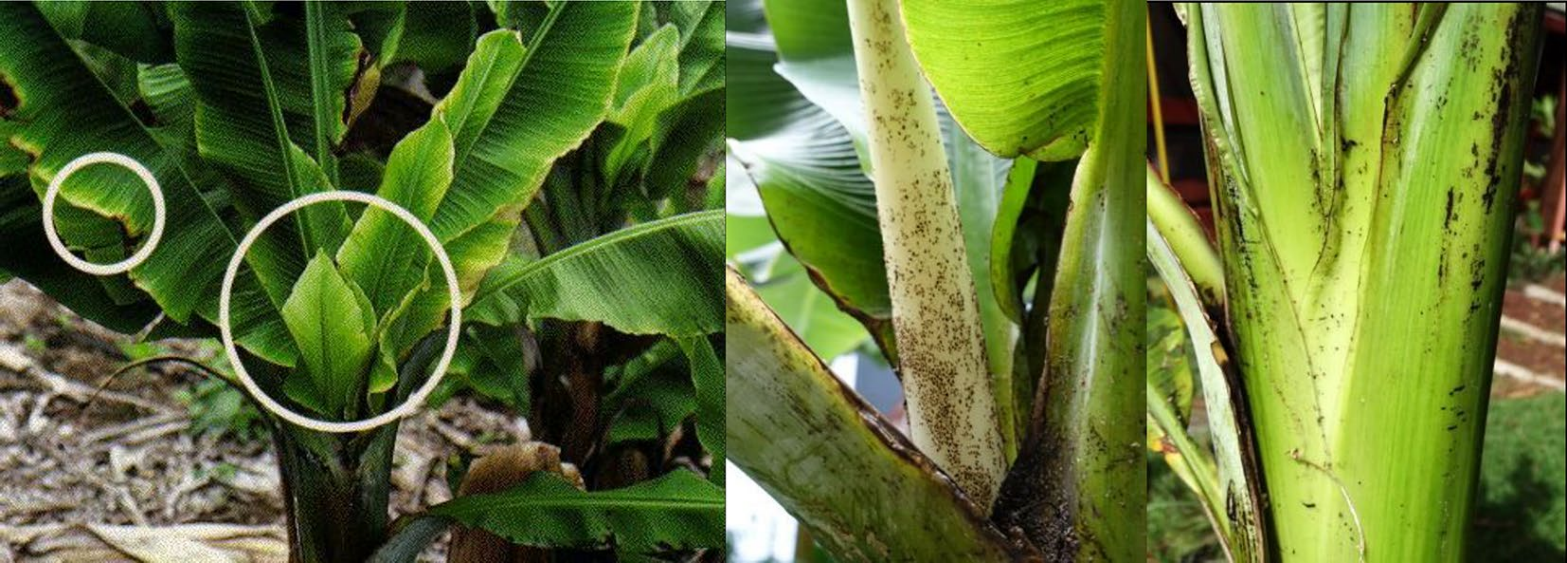
Bunchy top virus.

Banana Bunchy Top Virus (in Fig. 4), a viral agent that affects plants and belongs to the Nanoviridae family, causes extensive harm to banana plants, posing a notable risk to both the health of their leaves and the overall production of fruit. This unrelenting virus commences its attack by manifesting distinct symptoms, leaving an enduring imprint on the affected plant. The initial phase of infection is marked by the emergence of patches in the shape of dots and dashes along the lower portion of the midrib on the leaf. These patches gradually advance towards the leaf's veins, becoming more evident across the entire leaf blade, as depicted in Fig. 4^13,14^. The visual impact of these patches serves as a distressing indication of the escalating infection, signifying the relentless progression of the Banana Bunchy Top Virus. One of the most serious consequences of this viral onslaught is a significant decline in fruit production. The infected plants, which rarely bear fruit, experience a substantial decrease in productivity. Regrettably, the affliction caused by the Banana Bunchy Top Virus cannot be cured, underscoring the importance of prevention and early detection in order to minimize its impact on banana cultivation^2,15^.

### Banana streak

**Figure 5:**
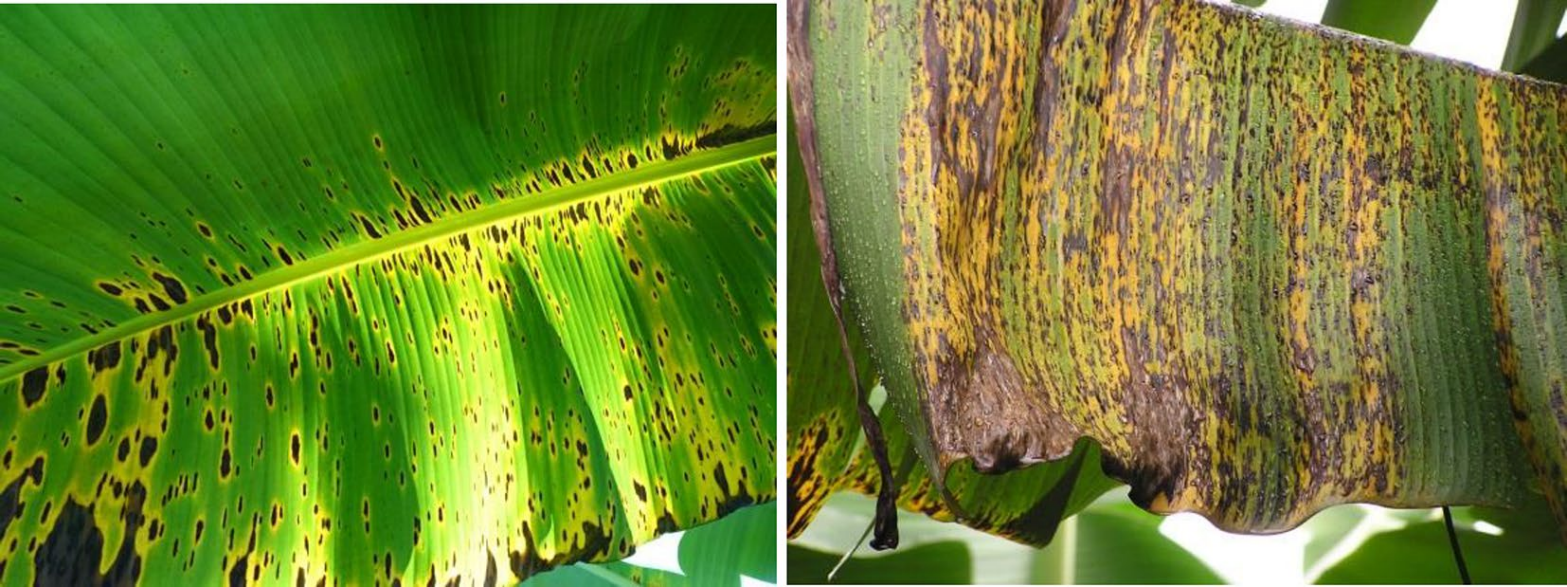
Banana streak disease.

Banana Streak (in Fig. 5), an affliction stimulated by the Banana Streak Virus (BSV), introduces an array of indications that fluctuate based on the plant variety, environmental factors, and particular virus strains. As this viral contamination takes root, distinctive visual indicators materialize, denoting the presence and progression of Banana Streak. Common indications of Banana Streak encompass yellow lines that traverse from the midrib to the margin of the leaf, as portrayed in Fig. 5^16^. These yellow lines are indicative of the viral influence, generating a visual arrangement that serves as a recognizable marker of the infection. In conjunction with the yellow lines, other indications may become apparent, introducing intricacy to the diagnosis. Fractured or connected chlorotic streaks may materialize, eventually intensifying in hue. As the ailment advances, mature leaves may showcase black streaking, symbolizing an advanced stage of Banana Streak. The repercussions of Banana Streak extend beyond mere visual distortion. The yellow lines and streaks impede the customary photosynthetic activity of the leaves, potentially impacting the overall health and productivity of the banana plant^17–21^.

### Infectious chlorosis

Infectious Chlorosis, which is primarily triggered by the Banana Cucumber Mosaic Virus, manifests a wide range of symptoms that significantly modify the visual aspect and vitality of the banana plants that are affected. The Banana Cucumber Mosaic Virus emerges as the principal culprit, giving rise to mosaic symptoms and distinctive streaked chlorotic bands in the leaves. Initially, the infected plants present mosaic symptoms that are characterized by broadly streaked chlorotic or yellow-green bands. This alteration in leaf coloration is a characteristic feature of infectious chlorosis. The leaves undergo a metamorphosis, becoming shorter and narrower compared to a normal leaf. Furthermore, the infected plants tend to exhibit a dwarfed stature, thus further underscoring the severity of the viral impact. The primary mode of transmission for infectious chlorosis is through the utilization of infected daughter suckers obtained from the diseased plant^17^. This method of dissemination highlights the significance of monitoring and controlling the use of plant propagules in order to prevent the spread of the Banana Cucumber Mosaic Virus. One of the notable characteristics of infectious chlorosis is the presence of light-yellow streaks that run parallel to the leaf veins. This pattern imparts a striped appearance to the affected leaves, thereby contributing to the visual intricacy of the disease. The following Table 1 brings the overview of various banana leaf diseases. In Table 1. We can see the summary of all the banana leaf disease mentioned in the previous section.

**Table 1:** Summary of banana leaf disease.

Sr. No.	Disease	Virus/fungus /bacteria	Early symptoms	Final stage
1	Panama Wilt	Fungus Fusarium Oxysporum	Leaves become brown or dark red and edges turns yellow	Leaves starts to hang down to the pseudo stem
2	Bract Mosaic	Bract Mosaic Virus	Stalks and midribs of the plant turn into spindle shape	Leaves turn brown
3	Black Sigatka	Phyllosticpina Nusarus	Grey spots on leaves and brown or black margin	Wide bands forms on either side of midrib
4	Bunchy top virus	Banana bunchy top virus (plant pathogenic virus)	Dot-dash patches over the lower edge of plant midrib	Dot-dash patches gradually progress towards leaf veins and leaf blades
5	Banana Streak	Streak (virus)	Yellow lines on midrib	Yellow lines on midrib
6	Infectious Chlorosis	Banana cucumber mosaic	Yellow and green bands on Leaves become short and narrow plant and plant also become dwarf	

## Related work

Manoj Patia and Vandhana Chaudary employed the K-means clustering technique and the SVM classifier, in conjunction with a texture feature method for extracting features. During the feature extraction process, the Gray Level Co-occurrence Matrix was utilized to compute various texture features. Metrics such as Contrast, Energy, and Correlation were quantified. They utilized a dataset of 80 images for sigatoka, out of which 65 were classified, resulting in an achieved accuracy of nearly 84%^22^. Antonio Criollo, Miguel Mendoza, Eduardo Saavedra, and Gustavo Vargas employed the CNN. The input layer takes an input of the form (300,300,3), where the first two coordinates represent the length and width of the image, and the last coordinate represents the number of channels. The accuracy attained ranged between 80 and 90% for 50 training samples^23^ (Criollo et al., instead of the authors' names). Eudarrado Correa, Melannie Garcia, Gustavo Grosso, Jose Huamantoma, and William Ipanaque initially processed the image through various stages such as data augmentation, segmentation, and Masking. In order to calculate the threshold value in the histogram equalization process, the Otsu method was employed, and the probability distribution of the pixels of the image was plotted. Subsequently, threshold values were computed using segments, and CNN was utilized for classification. After passing the image through Convolution and ReLu layers, the softmax function was applied, and the parameters were updated using gradient descent^24^.

**Figure 6:**
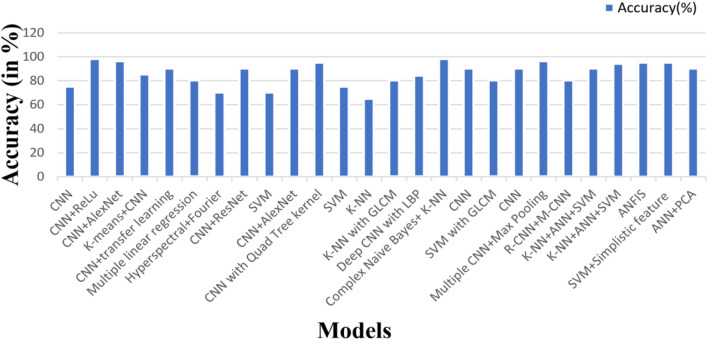
Calculated efficiency different machine learning algorithms^1^.

Ravindra Jogekar and Nandita Tiwari conducted a survey to determine the best algorithm among CNN, SVM, K-means, and KNN. The results revealed that CNN performed the best, but they also suggested that a combination of these algorithms would be the optimal choice for the disease identification process. Additionally, the calculated efficiency is depicted in Fig. 6^8^. The authors in^2^ described the Histogram of Oriented Gradients (HOG) feature extraction method. This method was utilized to generate the feature vector of a particular leaf image. The Random Forest classifier was employed for instance classification^25^. Furthermore, the authors concluded that logistic regression achieved the highest accuracy of 65.33%, while SVM achieved 40.33%, after comparing with other algorithms. This paper presents a CNN model for classifying banana leaf diseases^26^. CNN was implemented using RGB images of Banana leaf to detect plant diseases^18,27,28^. Three models were developed in this study using CNN. The first model was conducted without regularization, the second model utilized dropout, and the third model incorporated weight regularization techniques. The hyperparameters were optimized using the K-fold cross-validation method. Additionally, the Neural Network training was accelerated using the Adam optimizer. The results indicate that CNN without any regularization techniques proved to be the most suitable method for predicting banana leaf diseases.

The authors^29–31^ have proposed a deep learning model based on mobile technology for the detection of diseases affecting bananas. In order to test the effectiveness of the convolutional neural network (CNN) architecture, the authors have considered various models such as VGG, Resnet-18, Resnet-50, Resnet-152, and Inception V3. The training of the model has been carried out using TensorFlow. These five models have achieved different levels of accuracy, with VGG16 achieving 98.7%, Resnet 18 achieving 98.8%, Resnet 50 achieving 98.9%, Resnet 152 achieving 99.8%, and Inception V3 achieving 95.5%. For the purpose of mobile development, Inception V3 has been chosen due to its lower memory requirements and computational cost. The objective of the authors of these papers is to propose a system for predicting and rectifying diseases affecting banana leaves using a CNN. The prediction and rectification process involves two tasks^12,32^. The first task involves resizing the image to a standard input size, while the second task involves converting the image to grayscale in order to reduce the memory required for processing. Furthermore, feature extraction is carried out using CNN, and the classification is performed using the K-nearest neighbors (KNN) algorithm^18,33^.

The diseases of the banana plant are identified through the utilization of Hybrid convolutional neural networks^34,35^. The initial step in the process involves the pre-processing of the image, which entails resizing and the application of various image filtering techniques, such as median filtering, low pass filtering, and high pass filtering. In order to extract the desired features, the CNN architecture proposed in this paper is implemented. To classify the images, a two-step approach is employed, with the first step involving a binary SVM where the extracted features from CNN are utilized^36,37^. The second step involves the use of a multiclass SVM classifier to identify the specific type of disease. The methodology proposed in this paper yields an overall accuracy of 99% when compared to other deep learning techniques. A deep learning-based approach is implemented to identify diseases in banana leaves^38^. In the pre-processing stage, the authors resize the image to 60*60 pixels to enhance identification, followed by conversion to a grayscale image. As the authors utilize the CNN technique, the process involves convolution, pooling, and fully connected layers. Features are extracted through the convolution and pooling layers, while disease classification is carried out via the fully connected layers^39^. In Fig 6. We can see the different types of calculated efficiency different machine learning algorithms.

In this study, the authors employ the technologies of Close-range hyperspectral image and High resolution RGB image fusion. The resulting enhanced data is then utilized as input for the classifier. A K-nearest neighbor classifier is employed to classify the banana leaf^40,41^. The authors utilize an SVM classifier to identify the Portable sigatoka spot disease. The acquired banana leaf images undergo processing through reading, equalization, and segmentation techniques. After the completion of the pre-processing step, the images are then used to extract the features, enabling the identification of healthy and unhealthy leaves through color detection using the HSV color space. The extracted data is subsequently classified using SVM. The methodology proposed in this paper achieves an overall accuracy of 90%^27,42,43^. After conducting a comprehensive study or survey, it is observed that Image Acquisition, Image Pre-processing, Segmentation, Feature Extraction, Feature Selection, and Classification are employed in order to predict banana leaf diseases^44,45^. Additionally, it is noted from Table 2 that CNN and SVM classifiers play a significant role in classifying banana leaf diseases, contributing to more accurate results along with the algorithms used in segmentation, feature extraction, and feature selection.

**Table 2:** Types of machine learning algorithms considered for analysis.

[Ref]_years_	Description	K-means	Thresholding	HOG	SIFT	SVM	CNN	Random-forest
Devi et al. ^17^_2022_	Research on use of multiclass SVM for classifying the image					✓	✓	
Ravindra Jogekar ^8^_2022_	Study on Random Forest + ResNet for banana leaf disease identification							✓
Kavitha et al. ^3^_2022_	Study on CNN for classifying the banana leaf disease						✓	
Bharathi Raja and Selvi Rajendran^2^_2020_	Identification of the disease using K-means for segmentation	✓				✓		
Auti et al.^5^_2020_	Survey on various machine algorithms for banana leaf disease classification	✓				✓	✓	
Illahi and Mohammed^11^_2020_	Survey on CNN algorithm for disease identification						✓	
Aruraj et al.^16^_2020_	Study on use of SVM with Random Forest					✓		✓
Pai et al. ^13^_2020_	Study on 5-models of CNN for checking best model for image classification						✓	
Kohavi and John ^7^_2019_	SVM with LBP for classification of the image					✓		
^6^_2018_	Research on HOG method for feature extraction for banana plant image			✓				✓

## Methodology

**Figure 7:**
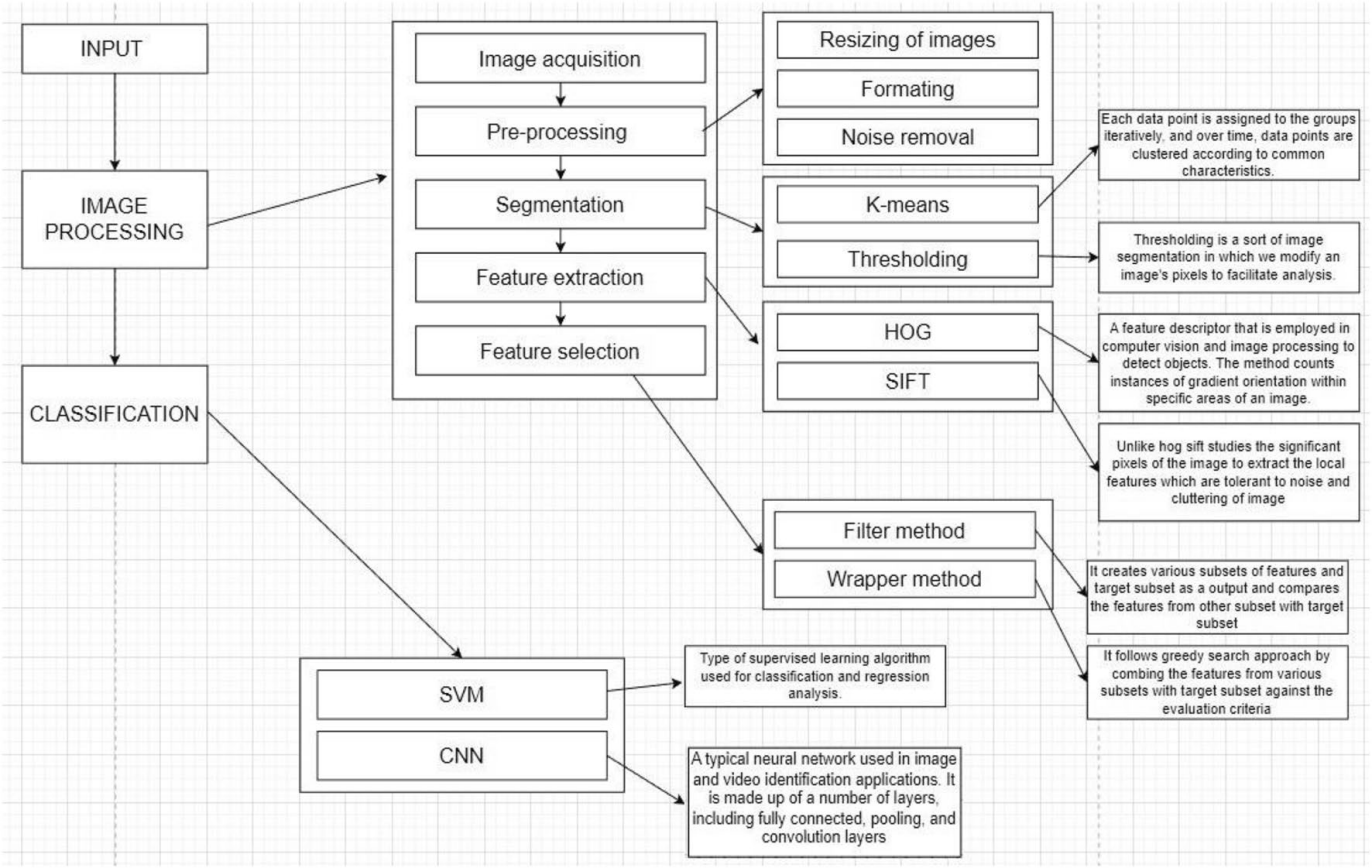
Processes involved in prediction^2,3,13^.

Banana exports in India during 2020–2021 exceeded INR 616 crores. As the year progresses, the number of banana plants also increases, resulting in higher demand for the fruit. Therefore, the aim is to cultivate more banana plants that are disease-free. Several authors have focused on the early detection and prediction of banana leaf diseases, outlining six major steps in the process. Figure 7 depicts the process of predicting banana leaf diseases.

### Image acquisition

Image acquisition is a crucial task that must be carried out before proceeding with any other processes. This phase involves capturing images with proper lighting, angle, and depth to ensure high resolution and quality. Capturing high-quality images ensures effective feature extraction in the subsequent phase. Additionally, it provides auto-exposure and auto-focus for image pre-processing^1,16^. After completing the image acquisition task, the next step is to preprocess the image by removing any noise present.

### Pre-processing

The pre-processing phase receives input from the image acquisition phase and performs minor modifications before sending the image to the next phase. The modifications involved in the process include resizing and formatting of images (SVG/PNG), as well as noise removal. The focus of the noise removal process is to identify defects and achieve greater accuracy^1,46^. After completing pre-processing and noise removal, the image input is sent to the segmentation layer to select the significant components of the image, making the subsequent task much simpler^1,3^.

### Segmentation layer

Segmentation receives input images from the pre-processing layer to select the shape and colour of the affected region, as well as the background color, among other factors^29^. This can be achieved using any of the following methods.

#### K-means clustering

This concept is implemented to identify the infected banana leaves by considering two distinct data points. The first data point corresponds to the highlighted portion, which is assumed to be the diseased section. The second data point corresponds to the dark portion, indicating the healthy portion^47^. The K-means clustering algorithm calculates the “centroids” of a set of N data points and computes the Euclidean distance. This process results in the formation of a cluster consisting of data points that are closer to a specific centroid. Once two distinct clusters are obtained from the data sets, the centroids of the clusters need to be recomputed. The process is then repeated by computing the Euclidean distance between all the data points and the new centroid. This iterative process of forming clusters continues until the centroids no longer undergo significant changes. The initial centroids are selected as K-objects, and nearby objects are assigned to the closest centroids^26,48^.

#### Thresholding

This methodology transforms the provided image, which may be in color or grayscale, into a simplified binary image through the suitable manipulation of pixels. By means of this conversion, the original image is partitioned into two distinct regions, specifically objects and background. If the intensity distribution of pixels in the image exhibits sufficient disparity, a single threshold value is sufficient for the entire image, known as a global threshold. In the context of a banana leaf image, in which f(x, y) represents a pixel containing a light object (the diseased portion) against a dark background, an appropriate threshold value, denoted as T, is diligently chosen to effectively discriminate between a diseased leaf and a healthy one by segmenting the intensity of the pixel. Dark portions correspond to healthier regions, while light portions indicate the presence of a diseased leaf^26^. The subsequent step involves the extraction of features, which is accomplished through the classification of all the aforementioned features.

$${\text{Let}}\,{\text{X}}\left( {{\text{i}},{\text{j}}} \right)\,{\text{be}}\,{\text{an}}\,{\text{image}}\,{\text{X(i,j) = }}\left\{ {\begin{array}{*{20}l} {0,} \hfill & {\text{p(i,j) < T}} \hfill \\ {1,} \hfill & {{\text{p(i,j)}} \ge {\text{T}}} \hfill \\ \end{array} } \right.$$

where p(i,j) is the pixel value at position (i,j), and T being the threshold value^26^.

### Feature extraction layer

After completing the segmentation process, the feature extraction phase studies the number of pixels in the image and captures significant information by identifying similar feature sets and distinguishing dissimilar ones. This process is also used to differentiate between healthy and affected banana leaf plants. To study the number of pixels in an image and extract the feature vector, follow the process outlined in^49^.

#### HOG feature (histogram of oriented gradients)

Histogram of Oriented Gradients (HOG) is a method used in image processing and computer vision for object detection^4^. It calculates features by identifying healthy regions using intensity gradients to distinguish affected regions. This approach operates on cells and is not affected by image transformations. Other methods, such as Hu moments, Haralick texture, and color histograms, may also be used in the process.

Hu moments are utilized to extract the outline of an object in the provided image. This is accomplished by converting the given image of a banana leaf from RGB to grayscale to highlight the outline of the leaf. Hu moments are then applied to characterize the pixels of the banana leaf in the image^1,2^.

**Algorithm 1:**
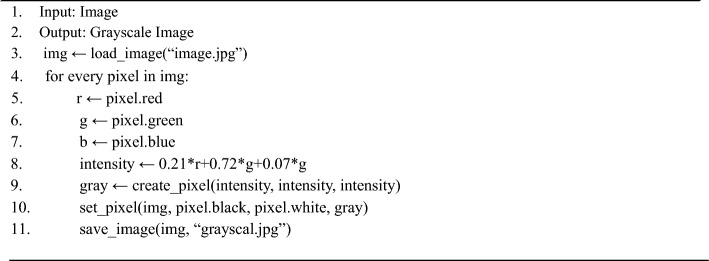
Hu Moments

Heralick texture features are functions of the Normalized Gray Level Co-occurrence Matrix (GLCM), which counts the co-occurrence of neighbouring gray levels in the given image. The GLCM is a square matrix that represents different aspects of the gray-level distribution in the Region of Interest (RoI). In predicting banana leaf disease, healthy and affected regions exhibit different textures. The process differentiates between the textures of healthy and affected regions in the banana leaf by utilising the adjacency matrix or GLCM matrix^1,49,50^.

The different texture features are given by^31^

1 $${\text{Range }} = { }\left[ {\left( {{\text{size}}\left( {{\text{GLCM}},{ }1} \right){ } - { }1} \right)} \right]^{2}$$

2 $${\text{Contrast }} = \mathop \sum \limits_{i,j = 0}^{N - 1} {\text{p}}\left( {{\text{i}},{\text{j}}} \right)\left| {{\text{i}} - {\text{j}}} \right|^{2}$$

(Where p(i,j) = pixel of the image)

3 $$\text{Correlation }=\sum_{i,j=0}^{N-1}\text{p}\left(\text{i},\text{j}\right)\left|\frac{\left(\text{i}-{\mu }_{i}\right)\left(j-{\mu }_{j}\right)}{\sqrt{{\sigma }_{i}^{2}{\sigma }_{j}^{2}}}\right|$$

4 $$\text{Energy}=\sum_{i,j=0}^{N-1}{p(i,j)}^{2}$$

5 $$\text{Homogeneity}={\sum }_{i,j=0}^{N-1}\frac{p\left(i,j\right)}{1+{\left(i-j\right)}^{2}}$$

7 $$\text{Entropy}=\sum p{\text{log}}_{2}p$$

(p = normalized histogram count).

The colour histogram technique is widely used to extract image colour features. Typically, RGB or HCV colour spaces are used, but for multispectral images, an N-dimensional colour histogram is used. However, due to the difficulty in representing N-dimensional histograms, RGB is often used by narrowing down the banana leaf into 256 colours to achieve maximum accuracy. This process results in the creation of four bins with varying intensities and intervals. The respective intensities of each bin are listed in Table 3.

**Table 3:** Bins and intensities.

Bins	Intensities
0	0–63
1	64–127
2	128–191
3	191–255

Finally calculate the pixel count in each bin to draw the color histogram for the banana leaf^1,2^.

**Algorithm 2:**
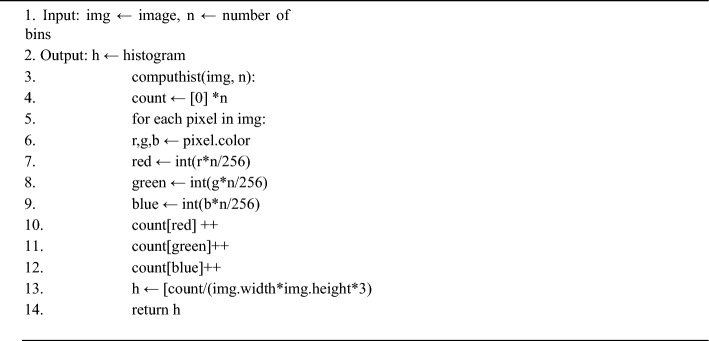
Color Histogram

#### Scale invariant feature transform (SIFT)

The Scale Invariant Feature Transform (SIFT) algorithm is designed to detect and describe local features, also known as 'key points', in an image. The SIFT process includes several phases: Space Generation, Difference of Gaussians (DoG), Key Point Detection, and Feature Description. Unlike the HOG method, the SIFT method only studies the significant pixels of the image to extract local features, making it more effective. This method identifies the important key points of both the affected and unaffected regions of the leaf. The key points are then grouped using k-means clustering or LS function. Various feature vectors are calculated for different clusters. The direction and magnitude of these key points are determined using neighboring pixels, and each key point is represented as a 128-dimensional feature vector^22,32^.

$${\text{D }} = \, \left\{ {\left( {{\text{xi}},{\text{ yi}}} \right)} \right\}$$

xi= feature vector; yi= particular class;

(class 1- Affected region class 2- Unaffected region)^32^.

During feature calculation, there is a possibility of mixing the features of affected and unaffected regions. To capture more accurate features, the Local Binary Pattern (LBP) method can be employed^13^. By combining the feature extraction and LBP methods, accuracy can be increased. The next step is to use the feature selection layer to choose the necessary features.

### Feature selection layer

The feature selection process aims to reduce the number of input variables, thereby decreasing computational costs and improving predictive model performance. This process involves three major types of methods: wrapper, filter, and embedded^30^. Wrapper methods include forward, backward, and stepwise selection. Filter methods can be carried out using ANOVA, Pearson Correlation, and Variance Thresholding. Embedded methods use Lasso, Ridge, and Decision Tree. However, the Filter method is preferred over the wrapper method due to its efficiency. The wrapper method employs a mining algorithm, whereas the filter method selects features before the mining process^30^. Since the wrapper method is computationally intensive, it is not suitable for large datasets. Therefore, the filtering method is a more practical choice^15^.

- *Filter method:* This method creates various subsets of features and target subset as output it is necessary to compare the features from each subset with the target subset. If any of the subset fits with the target subset then that subset alone is considered for further classification process^33^.
- *Wrapper method:* This method involves taking subset A and combining it with target subset T to train a new model. The process is then repeated with subsets A and B, and subsequently with other subsets as shown in Table 4. The resulting models are generated through this iterative process. After completing this step, we are left with several models. The objective is to identify the best model, which is done by backtracking through the models to find the best features. However, this process is timeconsuming, expensive due to the use of a large amount of data, and involves overfitting issues. Therefore, this method is not advisable for handling large amounts of data^15,33^.

**Table 4:** Wrapper method illustration.

A	B	C	D	T
A1	B1	C1	D1	T1
A2	B2	C2	D2	T2
A3	B3	C3	D3	T3
A4	B4	C4	D4	T4
·	·	·	·	·
·	·	·	·	·
An	Bn	Cn	Dn	Tn

Here A,B,C,D- feature subsets and T- target subset where.

A1,A2,A3……An are features of subset of A.

B1,B2,B3……Bn are features of subset of B.

C1,C2,C3……Cn are features of subset of C and.

D1,D2,D3……Dn are features of subset of D.

Recursive feature elimination:

Through the feature extraction process, we observe different feature vectors for different classes due to the existence of redundant feature vectors and in turn this reflects in classification output. Hence selection of optimal feature set from the features become mandatory. Finally, the selected features are given to the classification layer to correctly classify the image as diseased or healthy.

### Classification

Once all the foresaid methods have been completed their respective process the next step is to classify and to get a pure image of the banana leaf with accurate features vectors. The images are classified into affected or unaffected using any of the following classifiers^1,3^. For Image classification this research work considers the following classification algorithms.

#### Support vector machine

**Figure 8:**
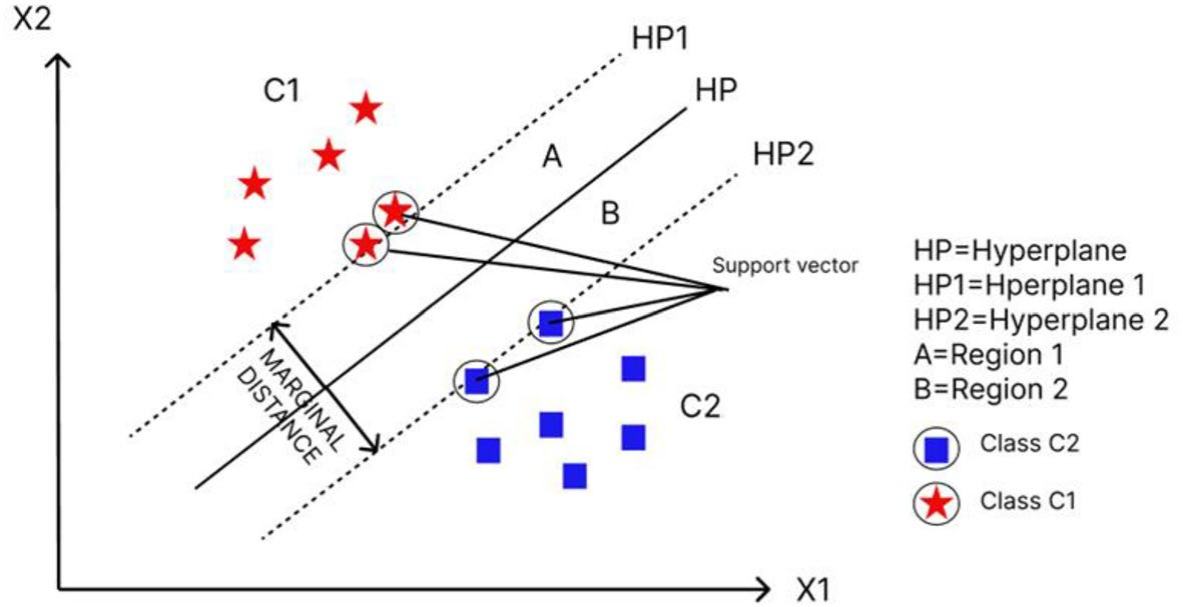
SVM illustration.

The Support Vector Machine (SVM) is a popular supervised machine learning algorithm used for classification and regression. The objective of the SVM algorithm is to classify data points in an N dimensional space by finding a hyperplane^14^. It is considered one of the best methods for both linear and nonlinear classification. The input data of the banana leaf is classified by SVM into two distinct classes: diseased and non-diseased. Two hyperplanes, HP1 and HP2, are drawn at the borders of the two classes (Fig. 8). The Euclidean distance between the hyperplanes is then calculated. Another hyperplane is drawn so that its maximum distance from HP1 and HP2 will be the classification boundary. Therefore, the equation for the classification boundary is determined.

7 $$(X) \, = wTx + {\text{ bg}}$$

Let us consider a random sample x1 illustrated in Fig. 8,

If g(x1) = wt*x1+b>0 then x1 belongs to C1

If g(x1) = wt*x1+b<0 then x1 belongs to C2

In^13^ cubic kernels are used to classify the leaf (Kernel is basically a function which transforms the training set into a nonlinear data set). The general solution used for classification is given by

8 $$(X) = (x_{i} ,x)$$

where {xi,yi} is the training set and K is the kernel function, with the value of yi as{− 1,1}^4,13,46^.

**Algorithm 3:**
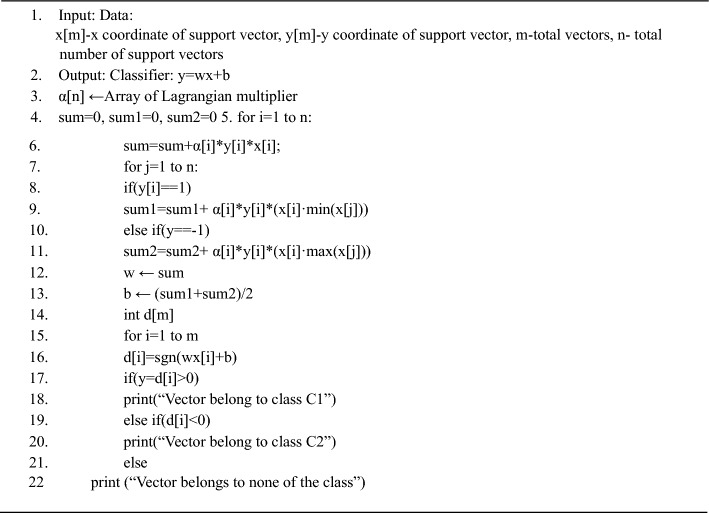
Support Vector Machine

#### Convolution neural network

Convolutional Neural Networks (CNN) are a popular deep learning architecture used for image recognition and processing^33,41^. They require a large amount of labelled data for training. The input is in the form of a triplet (x,y,z), where x and y represent the width and height pixel coordinates, and z indicates the number of channels. In the hidden layer, the image undergoes convolutional, pooling, and dense layers. The image first passes through the max pooling layer, followed by a convolutional layer, and then another pooling layer. The dimensions are flattened in the dense layer. CNN utilizes the ReLu and SoftMax activation functions^18,23,29^. The softmax function generates the probability vector of the output layer.

9 $$\text{P}\left(\rm{y}=\frac{\text{j}}{\rm{x}}\right)=\frac{{e}^{{(w}_{j}^{T}+{b}_{j})}}{\sum {e}^{{(w}_{j}^{T}+{b}_{j})}}$$

The parameters in the softmax function are updated using gradient descent algorithm which is given by:

10 $${w}{\prime}=w-\frac{\partial J\left(w,b\right)}{\partial w}$$

11 $${b}{\prime}=b-\frac{\partial J\left(w,b\right)}{\partial b}$$

where $\frac{\partial \text{J}(\text{w},\text{b})}{\partial \text{w}}$ is the slope in direction of w, and $\frac{\partial \text{J}(\text{w},\text{b})}{\partial \text{b}}$ is the slope in the direction of b^16^.

**Figure 9:**
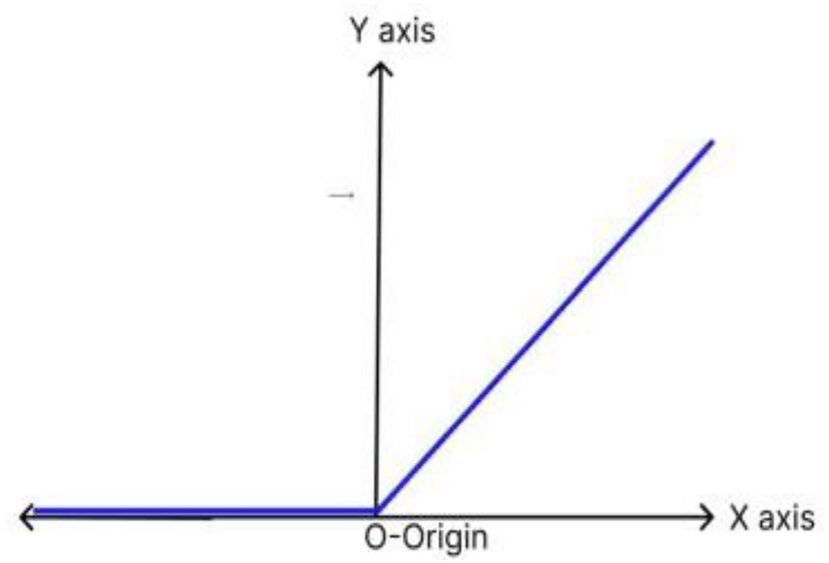
ReLu function.

The ReLu operation is piecewise linear function which is used to introduce non-linearity in the give dataset. After the convolution operation is performed, the pixel matrix has some negative values. To get rid of these values the ReLu operation is performed, by making all the values less than 0 and/or equal to 0 as shown in Fig. 9. The advantage of using ReLu activation function is achieved by not allowing the activation of all the neurons at same time. It is given by

$$f(x) = \left\{ {\begin{array}{*{20}l} x \hfill & {if\;x > 0} \hfill \\ 0 \hfill & {if\;x < 0} \hfill \\ \end{array} } \right.$$

**Figure 10:**
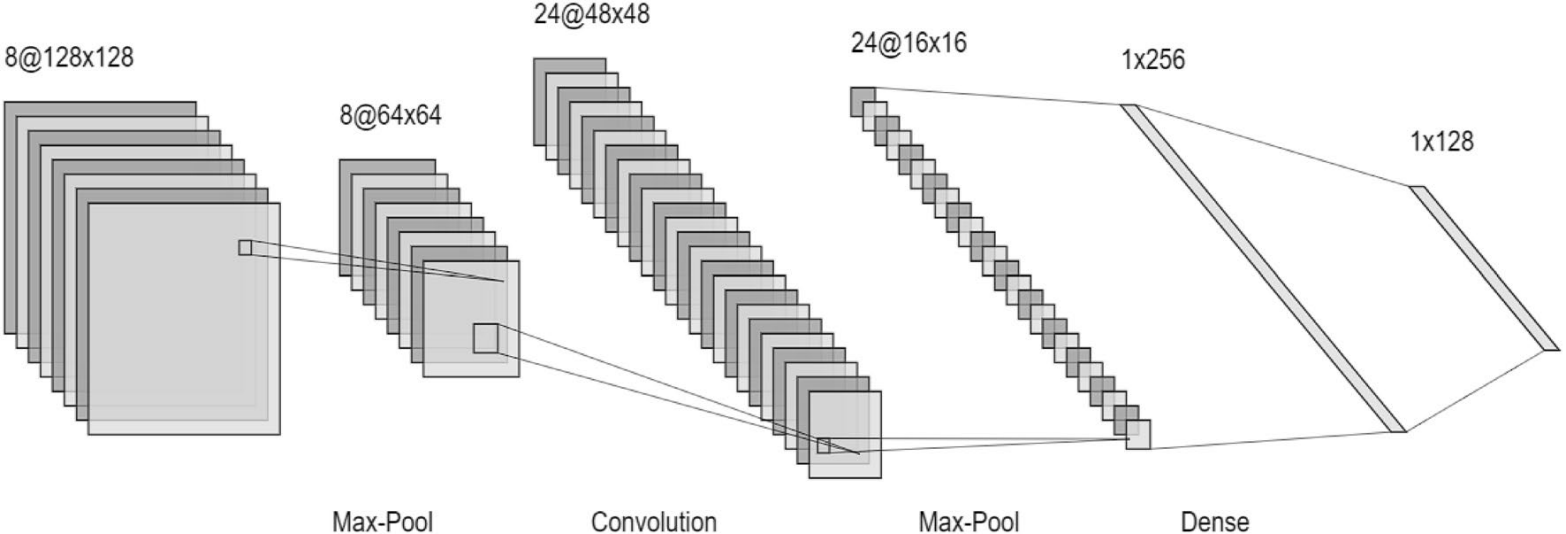
CNN illustration^3,25^.

ReLu operation is:

In the convolution layer the, firstly the features are taken from the feature extraction and the feature selection algorithms mentioned above. Then according to these features the banana leaf image is classified into different sections. The ReLu or softmax function removes the negative values^1,25^. The max pooling layer then tries to reduce the length of strides so that the computations become easy. For making the computations be more exact, a dense layer is added which reduces the dimensions. After all the operations are performed, next step is to classify the segments of the banana leaf into a diseased or a healthy leaf (Fig. 10). The limitation of CNN is that it cannot handle rotation^3,18,51^.

**Algorithm 4:**
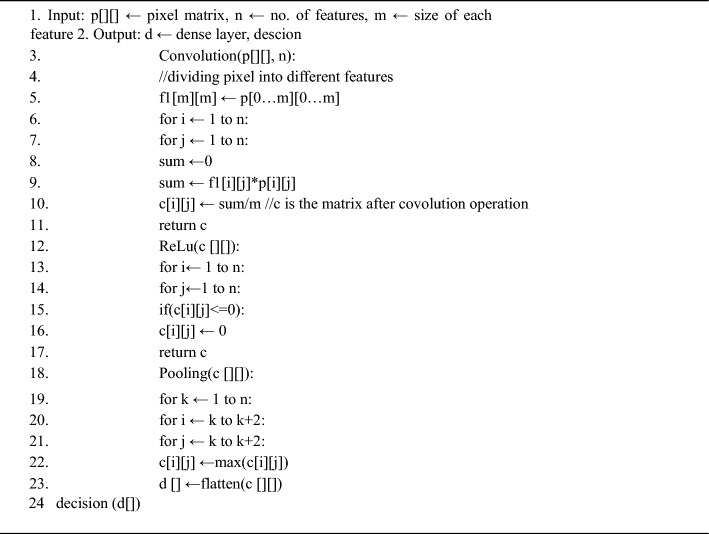
Convolution Neural Network

#### Artificial neural network

Artificial Neural Networks (ANN) is computational models inspired by the structure and function of biological neural networks in the human brain. They consist of interconnected nodes, or neurons, organized into layers. Information flows through the network from input nodes, through hidden layers, to output nodes, with each neuron performing a simple computation. ANNs are trained using a process called supervised learning, where they adjust the strengths of connections between neurons to minimize the difference between predicted and actual outputs. They excel at tasks such as pattern recognition, classification, regression, and decision-making, and have found widespread applications across various fields.

The input layer serves as the initial stage of the ANN, where extracted features from the images are transmitted for processing. Each feature extracted from the image corresponds to a neuron in the input layer, collectively forming the foundation for subsequent analysis.

Subsequently, the hidden layer(s) of the ANN undertake complex transformations on the input features, extracting relevant information crucial for accurate classification. These hidden layers act as intermediaries, orchestrating intricate mathematical operations to decipher the intricate patterns inherent in the input data. The design of the hidden layers, including the number of layers and neurons, is meticulously tailored to accommodate the complexity of the classification task and the nuances within the dataset.

Upon traversing through the hidden layers, the processed information converges onto the output layer, where final predictions or classifications are generated. In the case of banana leaf image classification, the output layer consists of neurons corresponding to distinct classes or categories of diseases prevalent among banana plants. For instance, neurons may represent fungal infections, bacterial infections, or nutrient deficiencies, depending on the classification schema.

Each neuron in the output layer employs an activation function, typically the softmax function for multi-class classification tasks, to produce probability distributions over the classes. This facilitates the interpretation of the network's output, indicating the likelihood of each class for a given input image. The combined efforts of the input, hidden, and output layers synergize to empower the ANN in accurately categorizing banana leaf images, thereby contributing to the effective management of diseases affecting banana plants.

## Performance analysis

The survey conducted on the identification of diseases affecting banana leaves has unveiled a significant gap in the current models, particularly regarding the treatment of scale and rotation invariance. The examined machine learning algorithms, such as random forest and decision trees, have demonstrated a relatively lower susceptibility to scale and rotation due to their independence from distances and angles between data points (Table 5). Nevertheless, there is still room for improvement to further enhance their resilience.

**Table 5:** Analysis of the machine learning algorithms.

Process	Algorithm	Strength	Weakness
Segmentation	K-means	Computationally very efficient and fast	Restricted mostly for spherical shape clustering
	Thresholding	Simplest image segmentation method	Difficult to find the threshold value
Feature extraction	HOG	No data loss as it directly works on the cell	Does not work for image rotation
	SIFT	Scale and rotation variant	A slow process
Classification	SVM	Works for linearly and nonlinearly separable classes	Supports only binary classification
	CNN	Works for different features instead of each pixel	Cannot handle rotation of the image

**Figure 11:**
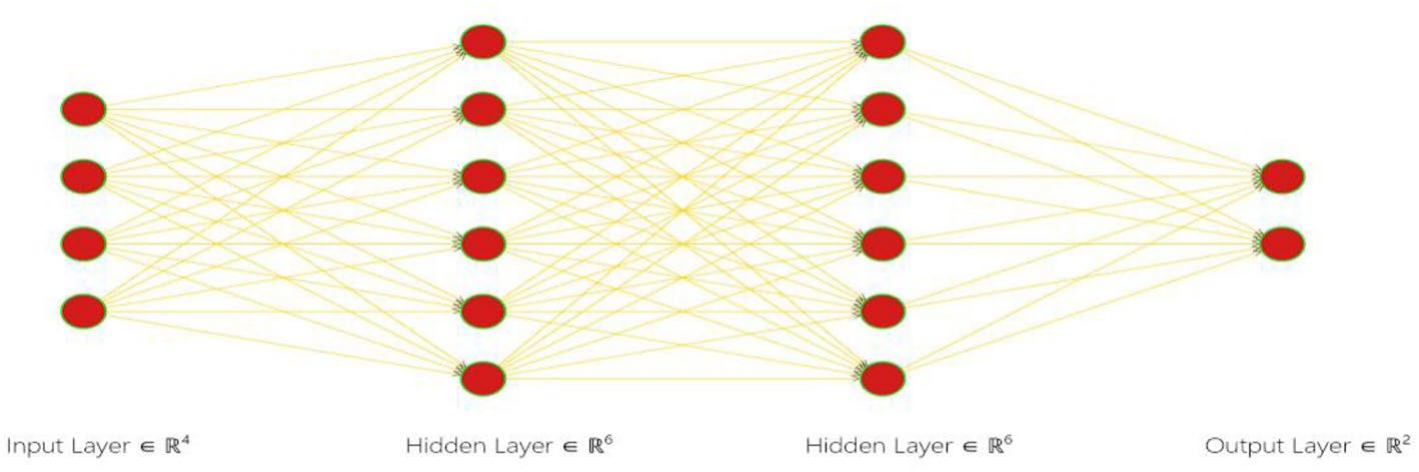
ANN architecture.

In order to tackle these challenges, the authors propose two innovative approaches aimed at enhancing both scale and rotation invariance. The first approach entails employing the Histogram of Oriented Gradients (HOG) technique for feature extraction and combining it with a Convolutional Neural Network (CNN) for classification (Fig. 11). HOG is selected for its capacity to handle scale invariance, while the incorporation of the Local Binary Pattern (LBP) algorithm guarantees invariance to rotation and scale. The second suggestion advocates for the utilization of the Scale-Invariant Feature Transform (SIFT) for feature extraction, along with CNN for classification, leveraging SIFT's inherent invariance to scale and rotation. These proposed approaches hold promise for addressing the limitations observed in the surveyed models^52^. By incorporating methods specifically designed to tackle scale and rotation, the authors strive to develop a more robust and efficient model for disease identification in banana leaves. The utilization of CNN in both approaches signifies a reliance on deep learning techniques, which have proven to be effective in image classification tasks.

**Figure 12:**
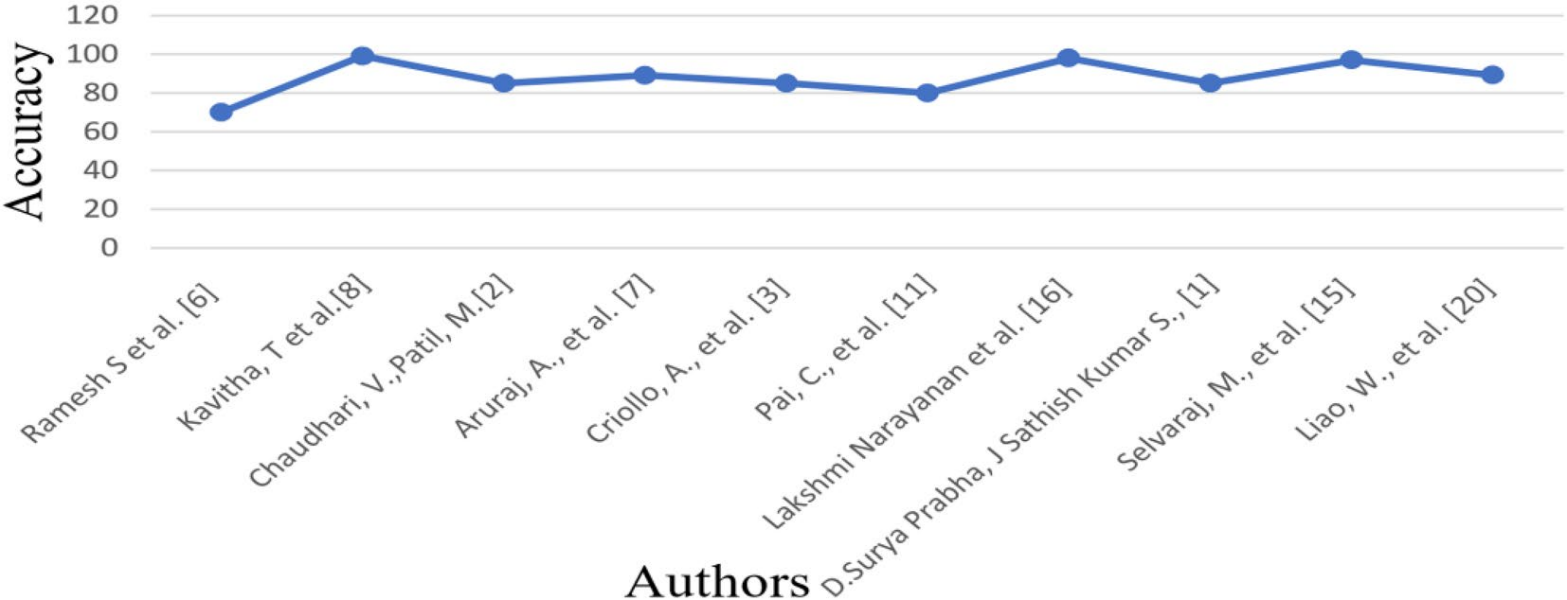
Graphical summary of the related work.

The graphical summary of the related work (Fig. 12) provides a visual depiction of the approaches discussed in the survey and underscores the proposed solutions. This visualization facilitates comprehension of the landscape of existing models and the direction the authors propose for future research. Table 6 delivers a comprehensive analysis of the articles examined in the survey, shedding light on the strengths and weaknesses of current methodologies. Looking forward, the authors suggest that incorporating datasets in video formats could serve as a potential avenue for detecting diseases in banana leaves. This recommendation underscores the evolving nature of data collection methods and emphasizes the necessity for adaptive machine learning algorithms capable of handling diverse data formats.

**Table 6:** Analysis through the articles considered in the survey.

Refs.	ML algorithm	Methodology	Result
^2^	Random Forest	Histogram of Oriented Gradients + Random Forest classifier (Classifier)	Model could classify images with 70% accuracy
^17^	Random forest	Random Forest + ResNet	The accuracy was 99.09%
^49^	SVM	K-means (Segmentation) + SVM (Classifier)	SVM had an overall accuracy of 85%
^13^	SVM	LBP(Feature extraction) + SVM + KNN as classifiers	The classifier had an accuracy of 89.1%
^23^	CNN	CNN (Classification)	The classifier was able to classify 50 images with a accuracy 80–90%
^25^	CNN	CNN used for classification	They reached a accuracy of around 80%
^15^	Multiclass SVM	CNN used for extracting features + Multiclass SVM (Classifier)	Average accuracy lies around 98% (BXW-98%, BFW-99%, BBTV-99%, BBS-97%)
^10^	SVM	Thresholding (Image segmentation) SVM(Classifier)	Reached overall accuracy of 85%
^46^	SVM	SVM + Random Forest	Accuracy is 97%
^7^	KNN	KNN(Classifier) plus Close-range hyperspectral image plus High Resolution RGB image	This model has accuracy of 89.21%

## Proposed models

In our approach, we are considering two distinct methodologies for disease identification/prediction on banana leaves: ANN (Artificial Neural Network) combined with SIFT (Scale-Invariant Feature Transform), and ANN combined with HOG (Histogram of Oriented Gradients) and LBP (Local Binary Patterns). This decision is rooted in the limitations and requirements specific to our problem domain.

While CNN (Convolutional Neural Network) models have demonstrated impressive accuracy in various studies, their lack of invariance to scale and rotation presents a significant drawback for our application. Additionally, CNNs cannot be seamlessly combined with feature extraction methods like SIFT or HOG + LBP due to their inherent design. CNNs operate by directly extracting features from raw image data, rendering them incompatible with techniques that rely on key points or histograms for feature extraction. Additionally, CNNs can be made invariant to scale and rotation however to achieve the same we will have to augment and add pooling layers to the CNN architecture which will make the CNN model as whole computationally more expensive. Moreover, the performance of CNN models depends on the size of the training dataset. Creating such a training dataset in agriculture and medical imaging is costly and time consuming. CNN's other drawback is black box nature thus making it difficult to explain the underlying principles of the model and to make predictions based on known data. Additionally, CNN models can be computationally expensive to train and use.

Although Recurrent Neural Networks (RNNs) excel in capturing temporal dependencies and sequential patterns, they are unsuitable for our banana leaf disease prediction task due to computational complexity. Banana leaf images lack inherent temporal dependencies, rendering RNNs inefficient and prone to computational overhead. Moreover, RNNs encounter issues like vanishing and exploding gradients, hampering training stability, especially in deep architectures.

Given these challenges and the non-sequential nature of our data, we favour alternative models such as ANN combined with SIFT or HOG + LBP to identify the diseases by providing more suitable solutions for classification. The following sections introduces the models and the implementation in detail of the foresaid models.

### ANN with SIFT model

The ANN with SIFT model harness the power of Artificial Neural Networks (ANN) in tandem with the Scale-Invariant Feature Transform (SIFT) algorithm. SIFT is renowned for its ability to detect scale-invariant key points and extract feature descriptors robust to changes in scale and rotation. By integrating SIFT with ANN, we leverage the strengths of both methodologies: the feature extraction capabilities of SIFT and the pattern recognition prowess of ANN.

#### Working principle

The process of extracting SIFT (Scale-Invariant Feature Transform) features and descriptors from images can be done using OpenCV involving several essential steps. Initially, the image can be loaded and converted from the default BGR (Blue, Green, Red) color space to HSV (Hue, Saturation, Value), offering flexibility for subsequent color thresholding operations. Then color thresholding can be performed to isolate specific colors within the image, followed by conversion to grayscale to simplify subsequent processing. Subsequently, a SIFT object is initialized to detect key points and compute descriptors. Leveraging the detectAndCompute() method of the SIFT object, key points representing distinct features are identified, and descriptors encoding the local appearance around each key point are computed. Computed descriptors can be filtered to retain a fixed number, ensuring computational efficiency. Descriptors are then flattened into a 1D array for streamlined handling. For visual inspection and testing, detected key points can be drawn on the original image. The resulting flattened descriptors, coupled with the key points, serve as inputs to ANN. This comprehensive process underscores the versatility and effectiveness of SIFT-based feature extraction technique in the ANN + SIFT model^53–55^

**Figure 13:**
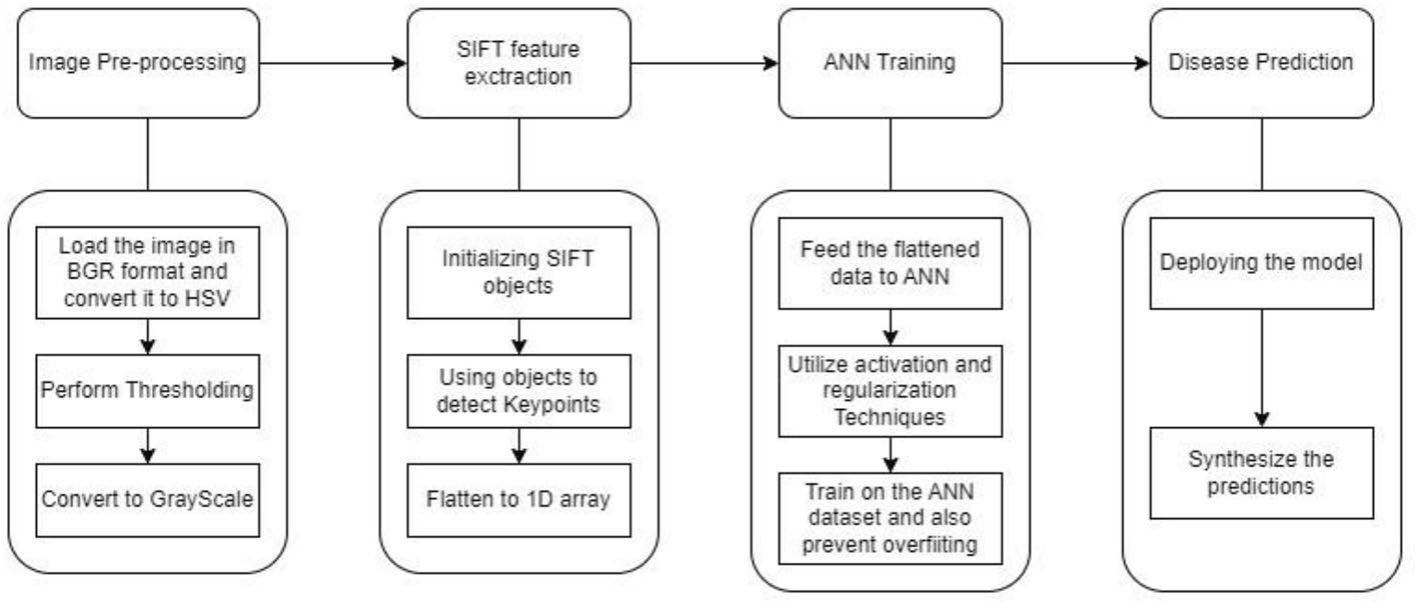
ANN + SIFT.

These descriptors which we got from the above method are then fed into an Artificial Neural Network (ANN). Within the ANN, the hidden layers can be employed using activation functions like ReLu,SoftMax and sigmoid to process the features extracted by SIFT. Additionally in order to check for overfitting of the artificial neural network regularisation techniques like L1 and L2 regularisation which involve adding a penalty term to the loss function based on the magnitudes of the model weights, Dropping out some percentage of neurons after each training iteration from the network as this method prevents individual neurons from relying too heavily on specific input features. These computations enable the network to discern complex patterns indicative of disease presence. Finally, the output layer synthesizes this information to provide actionable insights or classifications regarding the health status of the banana leaf. This seamless integration of SIFT-based feature extraction and ANN-driven analysis exemplifies a powerful pipeline for agricultural monitoring and disease identification/detection, with potential implications for advancing agricultural sciences and beyond. The combination of ANN and SIFT offers a synergistic approach to banana leaf disease prediction, providing resilience to scale and rotation variations while harnessing the discriminative power of neural networks (Fig. 13).

### ANN with HOG and LBP model

This approach adopts a complementary strategy by combining Artificial Neural Networks (ANN) with the Histogram of Oriented Gradients (HOG) and Local Binary Patterns (LBP) techniques. HOG captures local gradient orientations, highlighting textural information relevant to disease patterns on banana leaves, while LBP encodes texture information by comparing pixel neighbourhoods. These extracted features serve as rich representations of the underlying characteristics of banana leaf images. By integrating HOG and LBP with ANN, we create a robust framework capable of learning intricate patterns and relationships between features for accurate disease prediction. Notably, the HOG and LBP features exhibit invariance to scale and rotation. The robustness of HOG and LBP features to variations in lighting, scale, and orientation enhances the model's generalization capabilities, crucial for handling diverse conditions present in images of banana leaves. The synergy between ANN, HOG, and LBP significantly improves the model's ability to precisely classify disease states.

#### Working principle

The HOG + LBP + ANN approach for banana leaf disease prediction follows a multi-step process. Initially, raw images of banana leaves are preprocessed to enhance contrast, normalize pixel values, and resize them to a standard size. Subsequently, HOG (Histogram of Oriented Gradients) and LBP (Local Binary Pattern) features are extracted from the preprocessed images, capturing both global shape information and local texture patterns. The extracted features are then flattened into a 1D array, ensuring compatibility with the Artificial Neural Network (ANN) architecture. The ANN, configured with hyperparameters determined through cross-validation, is constructed to handle classification for disease prediction. The model undergoes stratified k-fold cross-validation, with learning curves visualized for each fold. After training, the model is evaluated on a separate test set, providing insights into its accuracy and performance metrics like confusion matrix and classification report. This comprehensive pipeline utilizes advanced image feature extraction techniques and a neural network to predict banana leaf diseases effectively.

**Figure 14:**
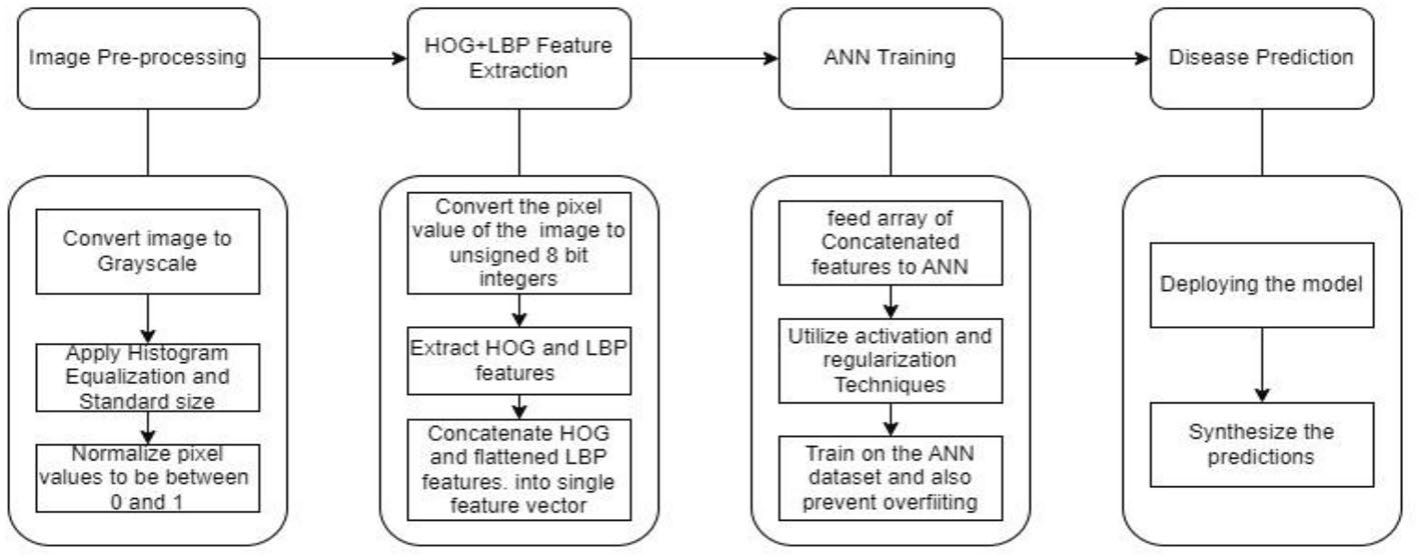
HOG + LBP + ANN.

The above extracted features are flattened and then passed to the ANN to enhance interpretability ensuring a consistent one-dimensional representation for each sample. This fusion allows the ANN to learn from a richer set of features, potentially enhancing the model's ability to recognize patterns associated with banana leaf diseases. Now we can normalize the combined feature vectors to ensure that it is invariant to variations in scale and rotation. This can involve techniques such as L2 normalization or Z-score normalization. This pre-processed data is then subjected to hyperparameter tuning, optimizing the neural network's architecture and parameters. The ANN, equipped with its adaptive learning capabilities, excels in recognizing intricate patterns associated with various leaf diseases (Fig. 14).

### Model improvements on search solution space

This section highlight, how the solution search space was improved with the use of the proposed models. SIFT (Scale-Invariant Feature Transform), HOG (Histogram of Oriented Gradients), and LBP (Local Binary Patterns) are feature extraction techniques that enhance the search solution space by capturing and representing key information from images. SIFT identifies distinctive features regardless of scale, rotation, or illumination changes. HOG focuses on the distribution of gradient orientations to capture shape information. LBP describes the texture by encoding local patterns. Together, these techniques provide a comprehensive and robust representation of images, allowing for more accurate and efficient search algorithms. Individually, SIFT, HOG, and LBP each contribute to enhancing the search solution space in their own unique ways. SIFT excels at capturing distinctive features regardless of scale, rotation, or illumination changes, making it highly effective for image matching. HOG, on the other hand, focuses on capturing shape information through gradient orientations, making it well-suited for object detection tasks. LBP, with its ability to encode local patterns, is particularly effective in describing texture and can be beneficial for texture-based image retrieval. ANN fused with the feature extraction will effectively outperforms in the disease detection, diagnosis and management with greater accuracy.

## Model training and implementation

In the two models proposed, this work implements SIFT models for feature extractors and artificial neural networks (ANNs). The performance of the proposed model was evaluated on BananaLSD (Leaf Spot Disease). The BananaLSD dataset contains annotated images of three categories of diseased banana leaves: Pestalotiopsis, Sigatoka, Cordana, and healthy banana leaves. In total, 937 images are contained in these four classes. Several argumentation techniques were applied to the original BananaLSD dataset to create a balanced training dataset. Several augmentation techniques were performed randomly on the original images to diversify the collection and negate the class distribution imbalance. There are 400 images in each augmented class. From the dataset, 70% of samples are used for model training and 30% for model testing. The Table 7 shows the recommended ANN model design parameters.

**Table 7:** ANN model design parameters.

	Value
Number of Input Neurons	700
Number of Hidden Layer	2
Number of Neurons in hidden layer	400
Number of output nodes	4
Learning rate	0.01
Momentum	0.95
Epochs	10
Regularization parameter	L1(0.7)

**Figure 15:**
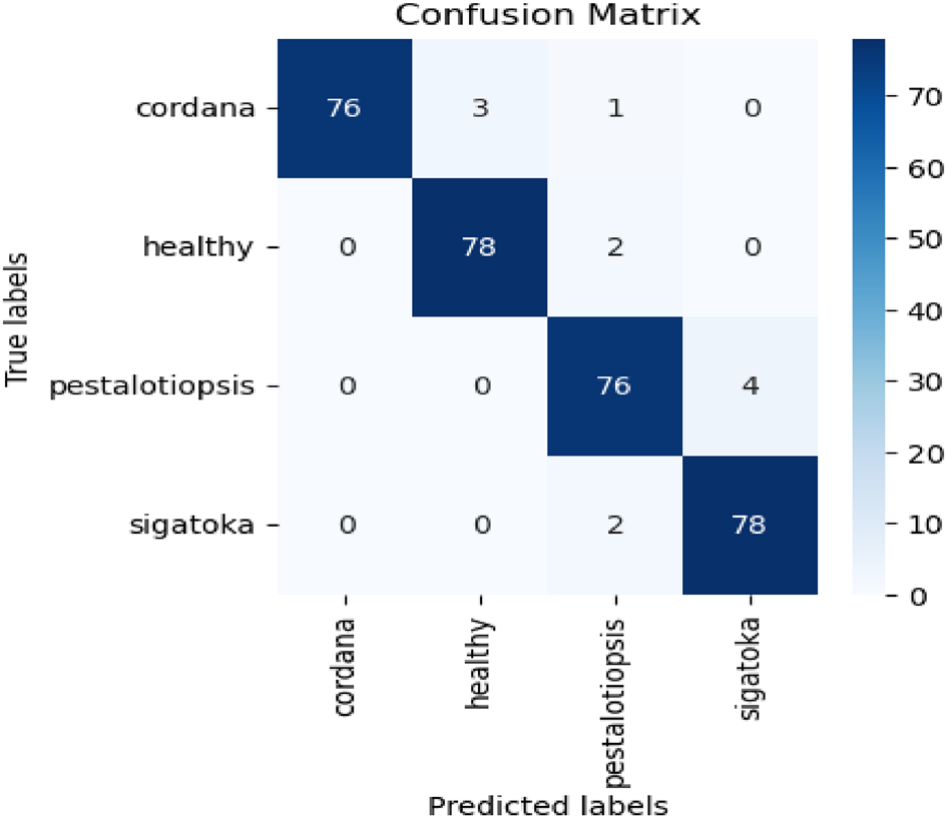
Confusion matrix for (SIFT + ANN).

Different trial and error methods are used to select the optimal design parameters. Categorical cross-entropy loss is adopted as a loss function to update the model training parameters as shown in Eq. 12.

12 $${L}_{CE}= -\frac{1}{N}\sum_{i=1}^{N}l\left({x}_{i}\right)\text{log}\left(p\left({x}_{i}\right)\right)$$

where $p\left({x}_{i}\right)$ probability of the image being correctly classified as class $l({x}_{i})$ and N is the number of training images. Accuracy is considered evaluation metrics to verify the performance of the proposed model. The confusion matrix of the proposed model is shown in Fig. 15.

**Figure 16:**
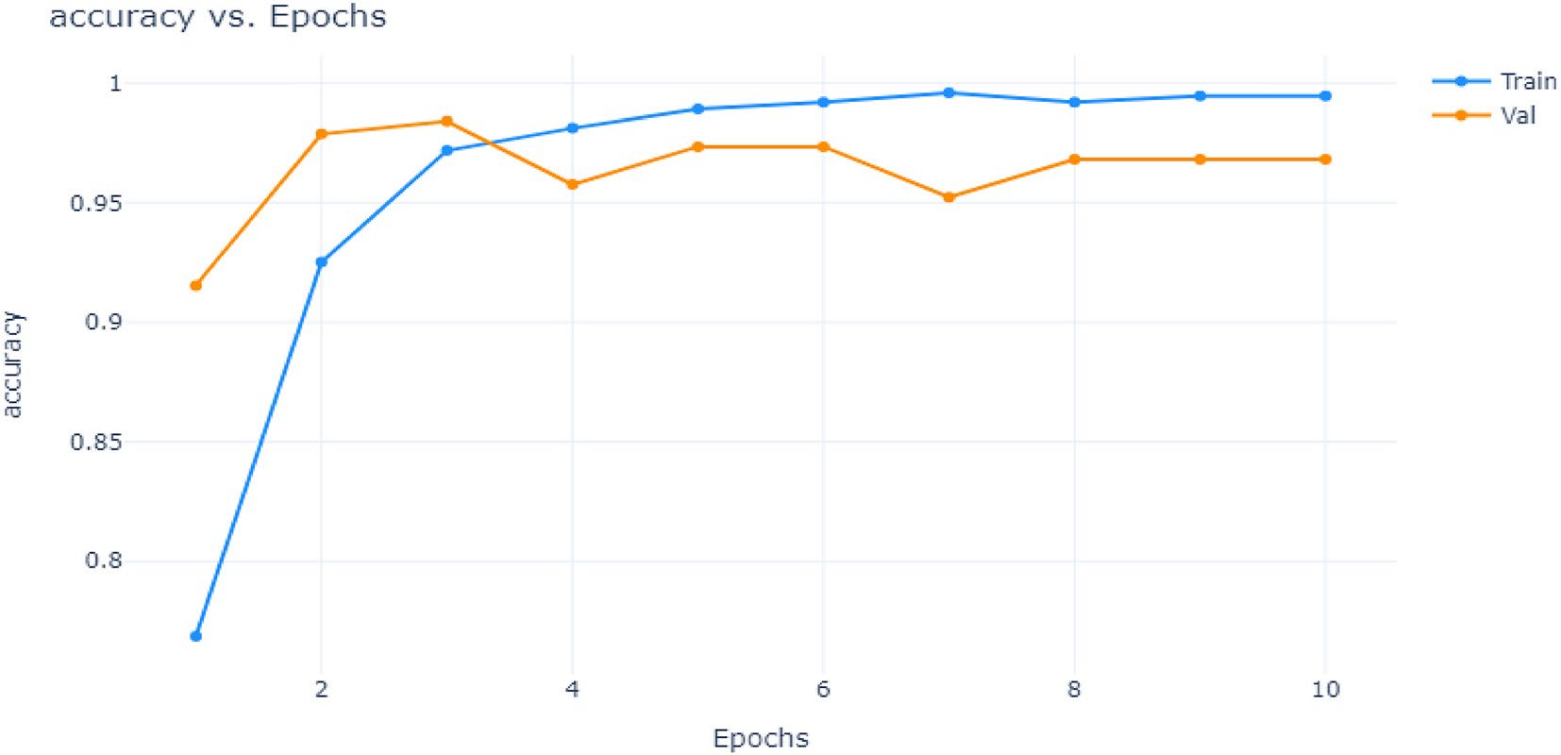
Accuracy vs Epochs.

**Figure 17:**
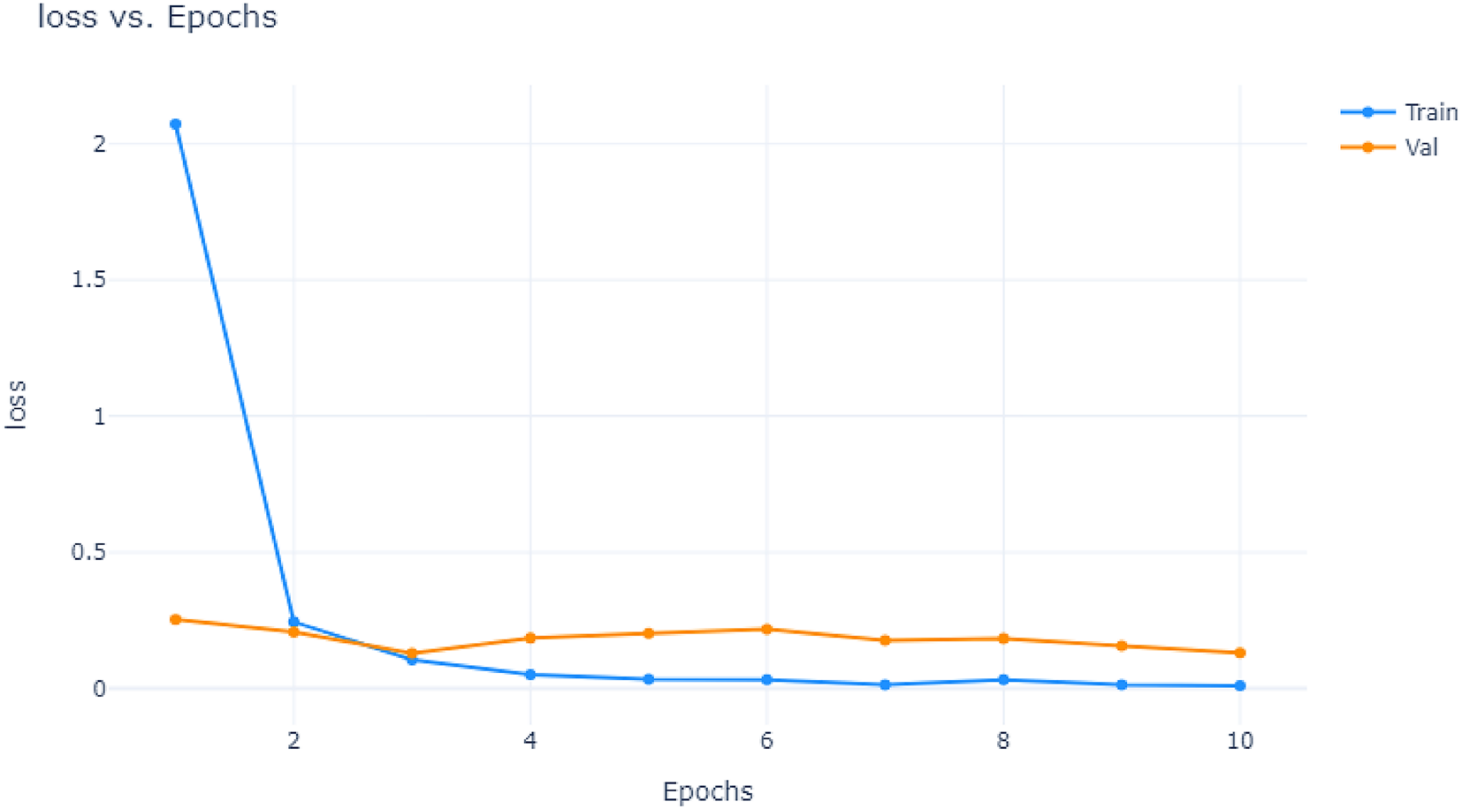
Loss Vs Epochs.

A comparison of the accuracy and loss values of the proposed model for different epochs is shown in Figs. 16 and 17. As can be seen, both accuracy and loss values improved with the number of epochs. This indicates that the model was able to learn and adapt as the number of epochs increased. This demonstrates that the model is an effective learning tool.

**Figure 18:**
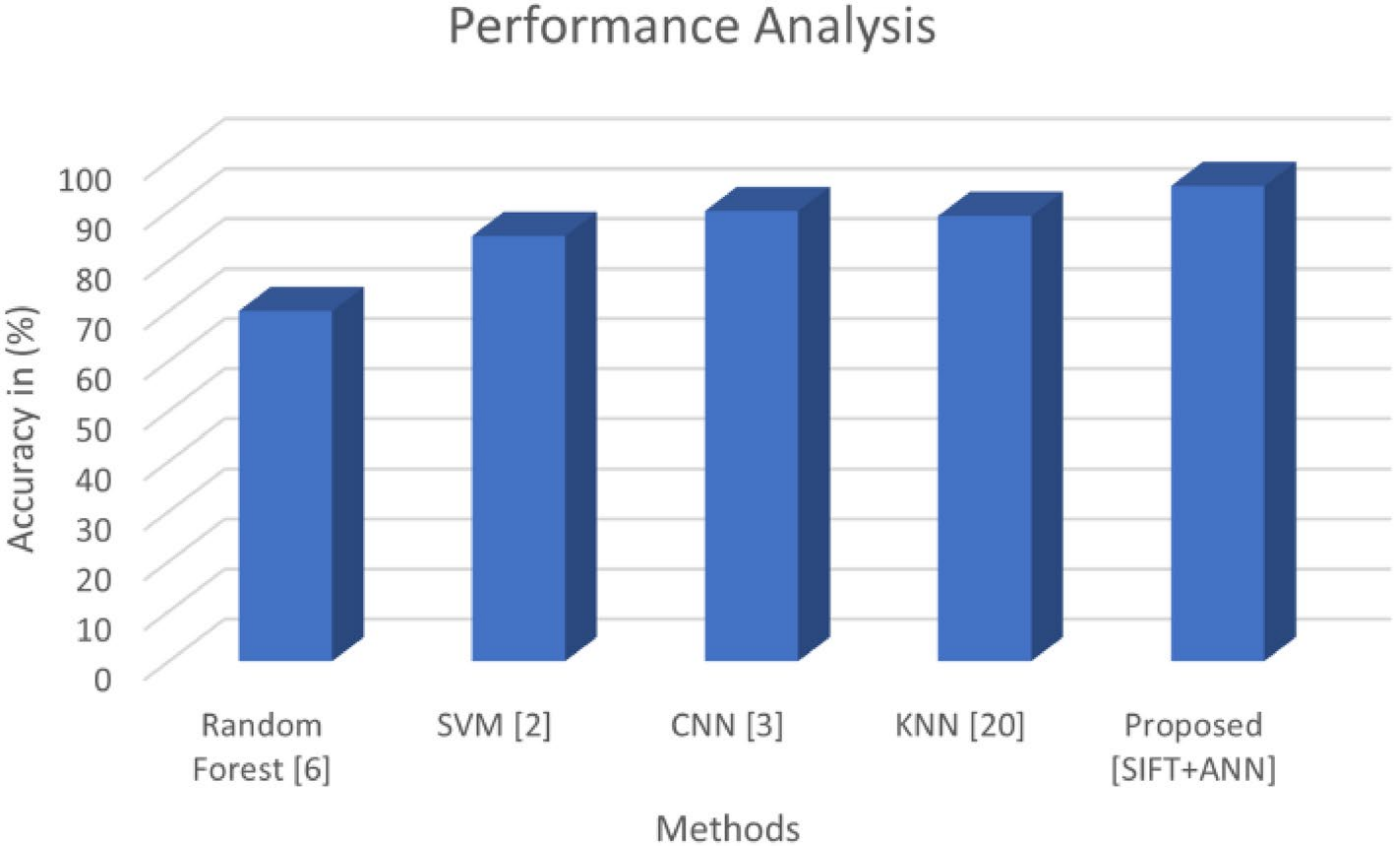
Quantitative analysis of the proposed model.

The quantitative performance of the proposed model is compared with the existing model which is discussed in the literature review section. Due to the effective feature representation of the SIFT feature extractor, the performance of the proposed classification model is significantly improved. The model takes advantage of the SIFT feature extractor, which can extract features that are more discriminative than those extracted by the traditional feature extractors. This has resulted in improved performance of the proposed model, allowing it to more effectively classify objects. The classification accuracy of the proposed model is 95% which is higher compared to the existing model. This shows that the proposed model is more reliable and efficient compared to the existing model (Fig. 18).

## Conclusion

The results of the survey conducted on the identification of the disease affecting banana leaves indicate that significant efforts are required to enhance the process of disease identification. The models discussed in the survey did not propose a solution that is rotation and scale invariant. Furthermore, there is currently no existing algorithm that is entirely immune to scale and rotation. However, algorithms such as random forest and decision trees are relatively less affected by scale and rotation due to their independence from distances and angles between data points for prediction purposes. This paper suggest two models namely, ANN with SIFT model and ANN with HOG and LBP model to identify the disease by mitigating the issue of rotation variations. Implementation results highlight the significance of the proposed ANN + SIFT model thus by achieving greater accuracy over the other models. Moreover, datasets in video formats could also be used to identify/detect banana leaf diseases by designing a suitable machine learning algorithm.

## Data Availability

The datasets used and/or analysed during the current study available from the corresponding author on reasonable request.

## References

[1] Auti NA, Kadam S, Satav S, Bhapkar P, Dhumal S (2018). Image Processing based Banana Leaf recognition and recommendation using machine learning. IJIRMPS.

[2] Bharathi Raja, N. & Selvi Rajendran, P. Comparative analysis of banana leaf disease detection and classification methods. IEEE Xplore (2022).

[3] Kavitha, T., Deepika, S., Nattaraj, K., Shanthini, P. & Puranaraja, M. Smart system for crop and diseases prediction using random forest and Resnet architecture. IEEE Xplore (2022).

[4] Narayanan, K. L., Santhana Krishnan, R., Harold Robinson, Y., Golden Julie, E., Vimal, S., Saravanan, V. & Kaliappan, M. Banana plant disease classification using hybrid convolutional neural network. *Hindawi Comput. Intell. Neurosci.* 1–13. 10.1155/2022/9153699 (2022). 10.1155/2022/9153699 PMC8890843 35251158

[5] https://plantix.net/en/library/plant-diseases/200019/banana-bract-mosaic-virus

[6] https://www.dpi.nsw.gov.au/biosecurity/plant/insect-pests-and-plant-diseases/banana-bract-mosaic-virus

[7] Kohavi R, John GH (1997). Wrappers for feature subset selection. Artif. Intell..

[8] Ravindra Jogekar Department of C, Tiwari, N. Summary of leaf-based plant disease detection systems: A compilation of systematic study findings to classify the leaf disease classification schemes. IEEE Xplore (2020).

[9] https://kisanvedika.bighaat.com/crop/sigatoka-disease-in-banana-causes-symptoms-preventive-measures- and https://kisanvedika.bighaat.com/crop/sigatoka-disease-in-banana-causes-symptoms-preventive-measures-and-management/management/

[10] Ahmad I, Hamid M, Yousaf S, Shah ST, Ahmad MO (2020). Optimizing pretrained convolutional neural networks for tomato leaf disease detection. Complexity.

[11] Illahi U, Mohammed G (2019). Monitoring and advanced control strategies in smart agriculture. IEEE.

[12] Sweetwilliams FO (2019). Detection of Sigatoka disease in plantain using IoT and machine learning techniques. J. Phys. Conf. Ser..

[13] Pai, C., Naik, D. & Sambhrama, B. Based banana leaf disease classification. *Int. Res. J. Eng. Technol. (IRJET)* **07**(04), 6120–6124 (2020).

[14] Kaur S, Babbar G, Gagandeep (2019). Image processing and classification, A method for plant disease detection. Int. J. Innov. Technol. Explor. Eng..

[15] Amara, J., Bouaziz, B. & Algergawy, *A. Deep Learning-based Approach for Banana Leaf Diseases Classification*, Lecture Notes in Informatics (LNI), Gesellschaft für Informatik, pp 79–88, Bonn (2017).

[16] Aruraj, A., Alex, A., Subathra, M. S. P., Sairamya, N. J., George, S. T. & Ewards, S. E. V. Detection and classification of diseases of banana plant using local binary pattern and Support vector machine. In *Proceedings of the 2019 2nd International Conference on Signal Processing and Communication (ICSPC)*, pp. 231–235, Coimbatore, India, March (2019). 10.1109/ICSPC46172.2019.8976582.

[17] Devi, R. D., Nandhini, S. A., Hemalatha, R. & Radha, S. IoT enabled efficient detection and classification of plant diseases for agricultural applications. In *Proceedings of the 2019 International Conference on Wireless Communications Signal Processing and Networking (Wisp NET)*, pp. 447–451, Chennai, India (2019).

[18] Hang J, Zhang D, Chen P, Zhang J, Wang B (2019). Classification of plant leaf diseases based on improved convolutional neural network. Sensors.

[19] Kaur S, Pandey S, Goel S (2019). Plants disease identification and classification through leaf images: A survey. Arch. Comput. Methods Eng..

[20] Pukale DD, Kokru G, Nadar S, Dhar S, Singh S (2019). A disease prediction and rectification system for banana leaf using CNN. Int. J. Sci. Dev. Res. (IJSDR).

[21] Malek, S., Bazi, Y., Alajlan, N., AlHichri, H. & Melgani, F. Efficient framework for palm tree detection in UAV images. IEEE xplore (2014).

[22] Patia, M. & Chaudary, V. Banana leaf disease detection using k-means clustering and feature extraction techniques. IEEE Xplore (2022).

[23] Criollo, A., Mendoza, M., Saavedra, E., Vargas, G. Design and evaluation of a convolutional neural network for banana leaf diseases classification. IEEE Xplore, August (2022).

[24] Guo Y, Zhang J, Yin C (2020). Plant disease identification based on deep learning algorithm in smart farming. Discret. Dyn. Nat. Soc..

[25] Louise, G., Tuazon, H., Duran, H. M. & Villaverde, J. F. Portable Sigatoka spot disease identifier on banana leaves using support vector machine. IEEE Xplore (2021).

[26] Freeman C, Kulic D, Basir O (2015). An evaluation of classifier specific filter measure performance for feature selection. Pattern Recogn..

[27] Wang Q, Qi F, Sun M, Qu J, Xue J (2019). Identification of tomato disease types and detection of infected areas based on deep convolutional neural networks and Object detection techniques. Comput. Intell. Neurosci..

[28] https://www.forestryimages.org/browse/detail.cfm?imgnum=5556347

[29] Beyene H, Joshi NA, Kotecha K (2018). Plant diseases prediction using image processing and machine learning techniques: Survey. Int. J. Comput. Appl..

[30] Liang W-J, Zhang H, Zhang G-F, Cao H-X (2019). Rice blast disease recognition using a deep convolutional neural network. Sci. Rep..

[31] https://apps.lucidcentral.org/pppw_v10/text/web_full/entities/banana_streak_disease_215.htm

[32] Selvaraj MG, Vergara A, Montenegro F, Ruiz HA, Safari N, Raymaekers D, Ocimati W, Ntamwira J, Tits L, Omondi AB, Blomme G (2020). Detection of banana plants and their major diseases through aerial images and machine learning methods: A case study in DR Congo and Republic of Benin. ISPRS J. Photogramm. Remote Sens..

[33] Li L, Zhang S, Wang B (2021). Plant disease detection and classification by deep learning-A review. IEEE Access.

[34] Lopez MA, Lombardo JM, Lopez M, Alvarez D, Velasco S, Terron S (2020). Traceable Ecosystem and strategic framework for the creation of an integrated pest management system for intensive farming. Int. J. Interact. Multimed. Artif. Intell..

[35] Azhar R, Tuwohingide D, Kamudi D, Suciati N (2015). Batik image classification using SIFT feature extraction, bag of features and support vector machine. Sci. Direct.

[36] Saleem MH, Potgieter J, Mahmood Arif K (2019). Plant disease detection and classification by deep learning. Plants.

[37] Liao, W., Ochoa, D., Zhao, Y., Rugel, G. M. V. & Philips, W. Banana disease detection by fusion of close range hyperspectral image and high-resolution RGB image. IEEE Xplore (2018).

[38] Selvaraj MG, Vergara A, Ruiz H (2019). AI-powered banana diseases and pest detection. Plant Methods.

[39] Unal, Z. Smart farming becomes even smarter with deep learning. IEEE **8** (2020).

[40] Sharath, D. M., Akhilesh, S. A., Rohan, M. G. & Prathap, C. Image based plant disease detection in pomegranate plant for bacterial blight. In *International Conference on Communication and Signal Processing, IEEE Advancing Technology of Humanity*, pp. 0645–0649 (2019).

[41] https://www.biisc.org/pest/banana-bunchy-top-virus/

[42] Kumari, C. U., Prasad, S. J. & Mounika, G. Leaf disease detection: Feature extraction with K-means clustering and classification with ANN. In *Proceedings of the Third International Conference on Computing Methodologies and Communication (ICCMC 2019)*. IEEE Xplore, pp.1095–1098 (2019).

[43] Yonow T, Ramirez-Villegas J, Abadie C, Darnell RE, Ota N, Kriticos DJ (2019). Black Sigatoka in bananas: Ecoclimatic suitability and disease pressure assessments. PLoS ONE.

[44] Ramesh, S., Hebbar, R., Niveditha, M., Pooja, R., Prasad Bhat, N., Shashank, N. & Vinod, P. V. Plant disease detection using machine learning. In *International Conference on Design Innovations for 3Cs Compute Communicate Control* pp 41–45. 10.1109/ICDI3C.2018.00017 (2018).

[45] Sanga S, Machuve D, Jomanga K (2022). Mobile-based deep learning models for banana disease detection. Eng. Technol. Appl. Sci. Res..

[46] Jayanthi, G., Archana, K. S. & Saritha, A. Analysis of automatic rice disease classification using image processing techniques. Int. J. Eng. Adv. Technol. **8**(3S) (2019).

[47] Prabha, D. S. & Satheesh Kumar, J. Study on banana leaf disease identification using image processing methods. IJRCSIT, ISSN No.: 2319–5010, **2** (2014)

[48] Shu W, Qian W, Xie Y (2022). Incremental neighbourhood entropy-based feature selection for mixed-type data under the variation of feature set. Appl. Intell..

[49] Anuraj, A., Alex, A., Subathra, M. S. P, Sairamya, N. J., Thomas George, S. & Vinodh Ewards, S. E. Detection and classification of diseases of banana using local binary pattern and support vector machine. IEEE Xplore (2019).

[50] Sreeji, C., Vineetha, G. R., Amina Beevi, A. & Nasseena, N. Survey on different methods of image segmentation. *Int. J. Sci. Eng. Res.* **4**, ISSN 2229–5518 (2013).

[51] Correa, E., Garcia, M., Grosso, G., Huamantoma, J. & Ipanaque, W. Design and implementation of a CNN architecture to classify images of banana leaves with diseases. IEEE Xplore (2020).

[52] Uwamahoro F, Berlin A, Bylund H, Bucagu C, Yuen J (2019). Management strategies for banana Xanthomonas wilt in Rwanda include mixing indigenous and improved cultivars. Agron. Sustain. Dev..

[53] Korkmaz S, Binol H (2018). Classification of molecular structure images by using ANN, RF, LBP, HOG, and size reduction methods for early stomach cancer detection. J. Mol. Struct..

[54] Jeyabharathi, J., Devi, S., Krishnan, B., Samuel, R., Anees, M. I. & Jegadeesan, R. Human ear identification system using shape and structural feature based on SIFT and ANN classifier. In *2022 International Conference on Communication, Computing and Internet of Things (IC3IoT)*, Chennai, India, pp. 01–06 (2022). 10.1109/IC3IOT53935.2022.9767893

[55] Sinkar, S. V. & Deshpande, A. M. Object recognition with plain background by using ANN and SIFT based features. In *2015 International Conference on Information Processing (ICIP)*, Pune, India, pp. 575–580 (2015). 10.1109/INFOP.2015.7489450

